# HPLC-ESI-HRMS/MS-Based Metabolite Profiling and Bioactivity Assessment of *Catharanthus roseus*

**DOI:** 10.3390/plants14152395

**Published:** 2025-08-02

**Authors:** Soniya Joshi, Chen Huo, Rabin Budhathoki, Anita Gurung, Salyan Bhattarai, Khaga Raj Sharma, Ki Hyun Kim, Niranjan Parajuli

**Affiliations:** 1 Central Department of Chemistry, Tribhuvan University, Kirtipur, Kathmandu 44618, Nepal; soniyajoshi157@gmail.com (S.J.); rabin.bc.992@gmail.com (R.B.); anitagurung9855@gmail.com (A.G.); khagarajsharma41@gmail.com (K.R.S.); 2 School of Pharmacy, Sungkyunkwan University, Suwon 16419, Republic of Korea; huochen_0213@163.com; 3 Paraza Pharma, Inc., 2525 Marie-Curie Avenue, Montreal, QC H4S 2E, Canada; salyanbiotech@gmail.com

**Keywords:** *Catharanthus roseus*, radical scavenging activity, antimicrobial assay, GNPS, secondary metabolites

## Abstract

A comprehensive metabolic profiling of *Catharanthus roseus* (L.) G. Don was performed using tandem mass spectrometry, along with an evaluation of the biological activities of its various solvent extracts. Among these, the methanolic leaf extract exhibited mild radical scavenging activity, low to moderate antimicrobial activity, and limited cytotoxicity in both the brine shrimp lethality assay and MTT assay against HeLa and A549 cell lines. High-performance liquid chromatography–electrospray ionization–high-resolution tandem mass spectrometry (HPLC-ESI-HRMS/MS) analysis led to the annotation of 34 metabolites, primarily alkaloids. These included 23 indole alkaloids, two fatty acids, two pentacyclic triterpenoids, one amino acid, four porphyrin derivatives, one glyceride, and one chlorin derivative. Notably, two metabolites—2,3-dihydroxypropyl 9,12,15-octadecatrienoate and (10*S*)-hydroxypheophorbide A—were identified for the first time in *C. roseus*. Furthermore, Global Natural Products Social Molecular Networking (GNPS) analysis revealed 18 additional metabolites, including epoxypheophorbide A, 11,12-dehydroursolic acid lactone, and 20-isocatharanthine. These findings highlight the diverse secondary metabolite profile of *C. roseus* and support its potential as a source of bioactive compounds for therapeutic development.

## 1. Introduction

Medicinal plants have long served as a vital source of pharmacological lead compounds, with their use dating back over 5000 years [1]. A large portion of the Global South still relies on herbal remedies to treat a range of illnesses, including skin diseases, malaria, diarrhea, dysentery, stomach pain, schistosomiasis, toothache, jaundice, and more [2,3,4]. Bioactive compounds derived from medicinal plants have garnered considerable interest due to their critical role in drug development, particularly in the treatment of infectious diseases, cancer, and cardiovascular disorders—often with minimal side effects [5].

*Catharanthus roseus* (L.) G. Don, commonly known as Madagascar periwinkle, is a rare undershrub in the Apocynaceae family. Traditionally used for its antidiabetic properties, this plant is now recognized as a rich source of bioactive constituents, producing over 100 alkaloids as well as other nitrogen-containing metabolites such as peptides, purines and their derivatives, amino acids, antibiotics, and amino sugars [6,7,8]. Among its notable compounds, indole alkaloids—found throughout the plant—play a major role in treating cancer, hypertension, diarrhea, diabetes, and Alzheimer’s disease, and have also shown potential in promoting wound healing [9,10,11].

Metabolic profiling is attracting increasing attention due to its potential in drug discovery, as it facilitates the analysis of biosynthetic pathways using the diverse bioactive compounds found in medicinal plants. Metabolomics is a technique that systematically analyzes large numbers of small molecules produced during cellular biochemical processes, revealing the unique chemical fingerprints associated with specific biological functions [12]. Metabolites, which are the end products of enzymatic activity, serve as accurate indicators of metabolic function [13]. There are two main approaches to metabolomic profiling: targeted and untargeted metabolomics. Targeted metabolomics focuses on the precise identification and quantification of specific metabolite groups within a sample, primarily to validate their presence [14]. In contrast, untargeted metabolomics aims to qualitatively and semi-quantitatively profile as many metabolites as possible in a single analysis [15]. Liquid chromatography coupled with mass spectrometry (LC-MS) is the preferred method for global metabolite profiling, as it enables the identification of a wide array of metabolites. Furthermore, Global Natural Product Social Molecular Networking (GNPS) is a web-based platform that clusters metabolites based on the similarity of their MS/MS fragmentation patterns, thereby enhancing metabolite annotation.

More than 130 terpene indole alkaloids (TIAs) have been identified in *C. roseus*, including vinblastine and vincristine—clinically important anticancer agents synthesized through the dimerization of vindoline and catharanthine [16]. In addition, *C. roseus* extracts have demonstrated antimicrobial, cytotoxic, and apoptotic activities, as evaluated by minimum inhibitory concentration (MIC), cell viability assays, and flow cytometry analysis, respectively—highlighting their potential in cancer therapy [17]. Moreover, *C. roseus* exhibits significant antioxidant properties, which can be assessed through assays such as 2,2-diphenyl-1-picrylhydrazyl (DPPH) radical scavenging, ABTS [2,2′-azino-bis(3-ethylbenzothiazoline-6-sulfonic acid)] radical scavenging, and ferric reducing antioxidant power (FRAP) [18]. In one study, Ferreres et al. employed high-performance liquid chromatography coupled with photodiode array detection and electrospray ionization tandem mass spectrometry (HPLC-DAD-ESI-MS/MS) to screen metabolites from various parts of *C. roseus*. They identified three caffeoylquinic acid derivatives and 15 flavonoids, marking the first comprehensive analysis of non-pigmented phenolic compounds in the species [19]. Similarly, five known indole alkaloids and three novel dimeric indole alkaloids were isolated from the whole plant of *C. roseus*. The absolute configurations and structures of the novel compounds were elucidated through nuclear magnetic resonance (NMR) spectroscopy and circular dichroism (CD) analysis [20]. Moreover, Yadav et al. evaluated the antibacterial activity of crude extracts obtained from the root, stem, and leaf of *C. roseus*. These extracts demonstrated inhibitory effects against *Bacillus subtilis*, *Staphylococcus aureus*, and *Escherichia coli* [21].

As part of our ongoing investigations into biologically active metabolites from diverse natural sources [22,23,24,25,26,27], this study aimed to separately evaluate the biochemical and biological activities of the leaf and stem extracts of *C. roseus*. The leaf extracts exhibited superior biological activity compared to the stem extracts. Based on this observation, we hypothesized that the leaves may contain more potent metabolites than the stems. To explore and identify the secondary metabolites responsible, we conducted HPLC-ESI-MS/MS analysis and GNPS analysis of the leaf extracts. Unlike previous studies that have focused primarily on targeted alkaloid screening or single-platform analyses, our work presents the first combined HPLC-ESI-HRMS/MS and GNPS-based untargeted metabolomic profiling of the methanolic leaf extract of *C. roseus*. This approach led to the annotation of 34 secondary metabolites, including two compounds, 2,3-dihydroxypropyl 9,12,15-octadecatrienoate and (10*S*)-hydroxypheophorbide A, reported here for the first time in *C. roseus*, and uncovered 18 additional metabolites via molecular networking. By integrating comprehensive bioactivity assays with advanced metabolomics, our study expands the chemical inventory of *C. roseus* and provides new leads for therapeutic development. Here, we present the bioactivity assessment of *C. roseus* extracts and HPLC-ESI-HRMS/MS-based metabolite profiling.

## 2. Results

### 2.1. DPPH Radical Scavenging Assay

The collected stems and leaves of *Catharanthus roseus* were air-dried, crushed, powdered, and extracted using the maceration technique with four different solvents: methanol, ethyl acetate, dichloromethane (DCM), and hexane. Leaf extracts prepared using methanol, ethyl acetate, DCM, and hexane were labeled as A01, A02, A03, and A04, respectively, while stem extracts prepared with methanol and ethyl acetate were designated A05 and A06, respectively.

The DPPH radical scavenging activity of the *C. roseus* extracts ranged from 53.12 ± 1.60 to 94.12 ± 1.03 µg/mL. Quercetin, used as the positive control, exhibited an IC_50_ value of 3.89 ± 0.68 µg/mL. All extracts demonstrated lower scavenging activity compared to quercetin, as shown in Table 1. The IC_50_ value of the methanolic leaf extract (A01) reported in a previous study was 49.74 ± 0.52 µg/mL, which is comparable to our result, with a difference of approximately 4 µg/mL [28]. In contrast, the stem extracts (A05 and A06) showed a weaker scavenging effect, even at higher concentrations, as illustrated in Figures S1 and S2, suggesting their relatively lower DPPH scavenging potential compared to the leaf extracts. A two-way ANOVA with replication indicated that all comparisons were statistically significant (*p* < 0.05). A subsequent Tukey’s HSD post hoc test revealed that the scavenging activity of the hexane extract differed significantly from those of the methanol, ethyl acetate, and dichloromethane extracts (*p* < 0.05). Detailed results are presented in Supplementary Tables S5 and S6.

### 2.2. Antimicrobial Assays

**Figure 1:**
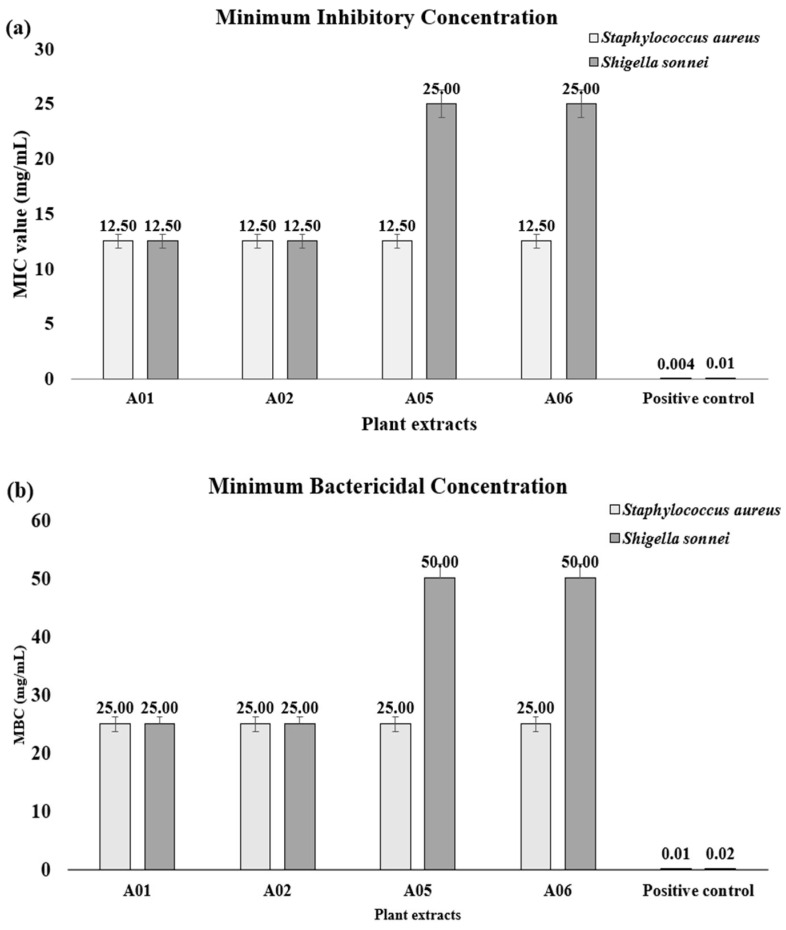
(**a**) MIC values and (**b**) MBC values of different *C. roseus* extracts against *Staphylococcus aureus* and *Shigella sonnei*. A01 and A02 represent the methanolic and ethyl acetate extracts of the leaves, respectively, while A05 and A06 represent the methanolic and ethyl acetate extracts of the stem, respectively. Neomycin was used as the positive control. All data are presented as mean ± standard deviation (n = 3).

The antimicrobial activity of the plant extracts (A01–A06) was evaluated against various Gram-positive and Gram-negative bacteria, including *Staphylococcus aureus*, *Shigella sonnei*, *Klebsiella pneumoniae*, and *Escherichia coli* (Table S1). Among all extracts, the methanolic leaf extract (A01) and the methanolic stem extract (A05) demonstrated the mild levels of inhibition against *S. aureus* and *S. sonnei*, weak inhibition against *K. pneumoniae*, and no detectable inhibition against *E. coli*. However, they exhibited lower activity than the positive control (neomycin). Accordingly, the minimum inhibitory concentration (MIC) and minimum bactericidal concentration (MBC) of the methanolic and ethyl acetate extracts from both leaves and the stem (A01, A02, A05, and A06) were determined against *S. sonnei* and *S. aureus* (Figure 1). The methanolic (A01) and ethyl acetate (A02) extracts showed MIC values of 12.5 mg/mL and MBC values of 25 mg/mL against both bacterial strains. In comparison, A05 and A06 exhibited higher MIC and MBC values (25–50 mg/mL), indicating lower potency. The positive control (neomycin) showed significantly lower MIC and MBC values, with MICs of 0.004 mg/mL and 0.01 mg/mL and MBCs of 0.01 mg/mL and 0.02 mg/mL for *S. sonnei* and *S. aureus,* respectively, confirming the validity of the assay (Table S2, Figures S1–S6).

### 2.3. Brine Shrimp Lethality Assay

The biological activity of methanolic leaf extract (A01) of *C. roseus* was evaluated using a brine shrimp lethality assay against newly hatched *Artemia salina* nauplii. The lethality of the methanolic leaf extract (A01) was assessed by calculating mortality percentages based on the number of dead nauplii exposed to different extract concentrations, as summarized in Table S3. The results showed a concentration-dependent increase in mortality, with higher extract concentrations causing greater lethality. All nauplii died in the positive control group, while 100% survival was observed in the negative control. The methanolic leaf extract (A01) exhibited toxicity, with an LC_50_ value of 914.98 µg/mL, suggesting weak anticancer properties. However, this assay provides only preliminary evidence. Therefore, an MTT assay was subsequently conducted to further evaluate the anticancer potential of the methanolic leaf extract.

### 2.4. Cytotoxicity Assay

**Figure 2:**
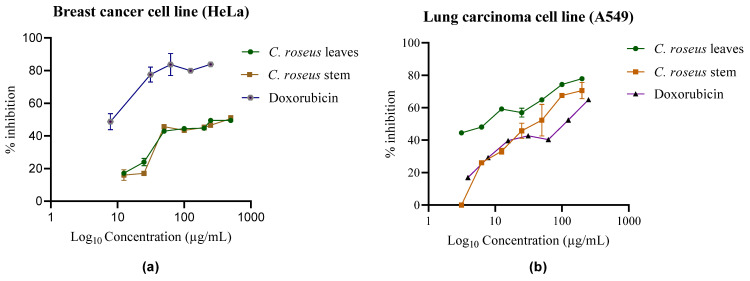
Cytotoxic activity of methanolic extracts of *C. roseus* and doxorubicin against (**a**) HeLa and (**b**) A549 cell lines. All data are presented as mean ± standard deviation (n = 3).

The MTT assay was conducted on lung carcinoma cell lines (A549) and cervical cancer cell lines (HeLa cell lines) to evaluate the cytotoxic effects of methanolic extracts (A01 and A05) from *C. roseus*. IC_50_ values were calculated to determine the concentration required to inhibit 50% of cell viability (Figure 2). The methanolic extracts from the stem and leaves exhibited IC_50_ values of 32.5 ± 0.01 µg/mL and 37.5 ± 0.10 µg/mL, respectively, against HeLa cells. Similarly, against A549 cells, the methanolic leaf and stem extracts showed IC_50_ values of 35.98 ± 0.89 µg/mL and 31.64 ± 0.12 µg/mL, respectively. As shown in Figure 2, increasing the extract concentration resulted in higher inhibition rates, leading to decreased cell viability. These results suggest that *C. roseus* extracts possess moderate cytotoxic potential against both cell lines. Additionally, the RealTime-Glo™ (Promega Corporation, Madison, WI, USA) MT Cell Viability Assay performed on the HeLa cell line revealed IC_50_ values of 45 ± 0.15 µg/mL and 40 ± 0.15 µg/mL for both the leaf and stem methanolic extracts, respectively (Figure S44). Since the assay substrate is reduced only by metabolically active cells, a decrease in luminescence with increasing extract concentration reflects reduced cell viability. Doxorubicin (IC_50_: ~15.26 ± 0.91 µg/mL) was included as a positive control to evaluate the assay’s robustness and dynamic range.

### 2.5. Metabolite Profiling Using HPLC-ESI-HRMS/MS

**Figure 3:**
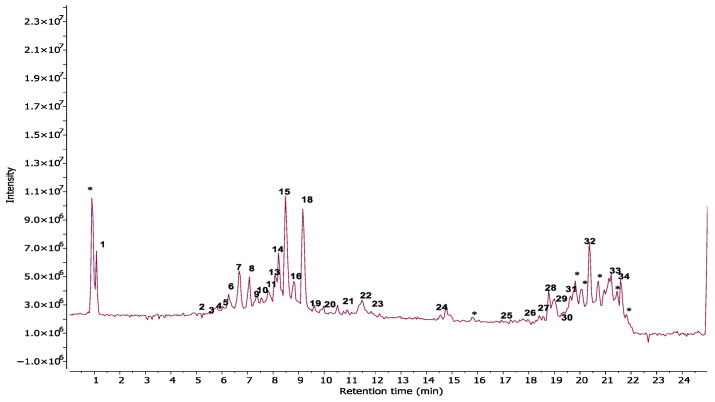
Total ion chromatogram (TIC) of the methanolic leaf extract (A01) of *C. roseus* obtained via HPLC-ESI-HRMS/MS in positive ion mode, showing annotated metabolites. (* Unidentified peaks).

**Figure 4:**
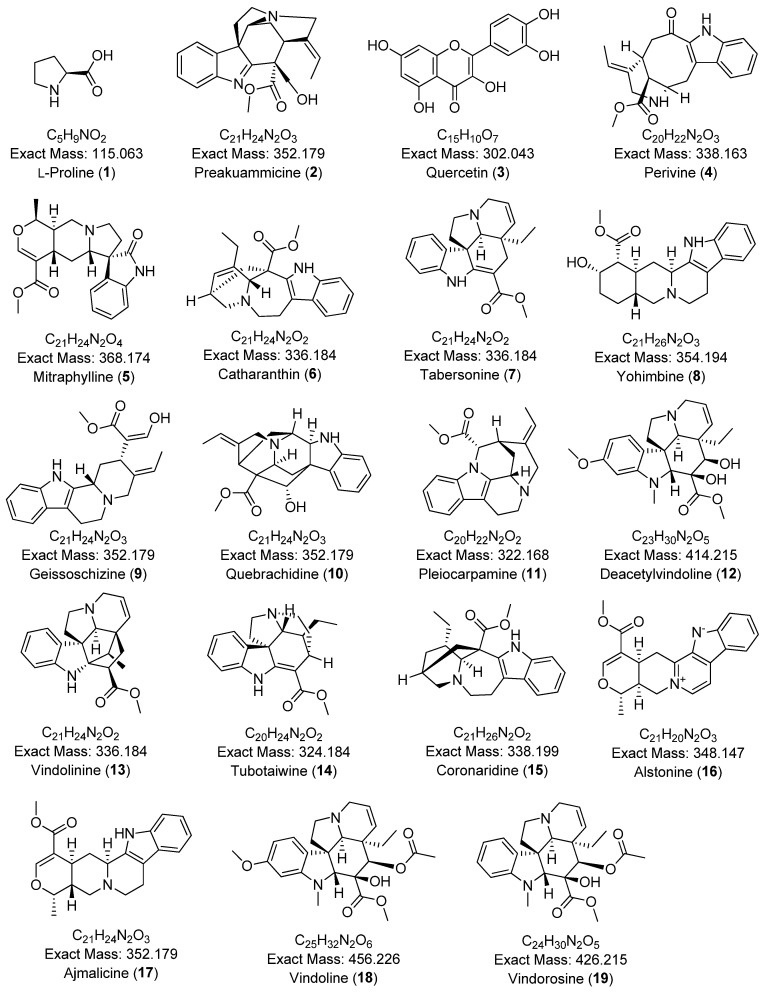

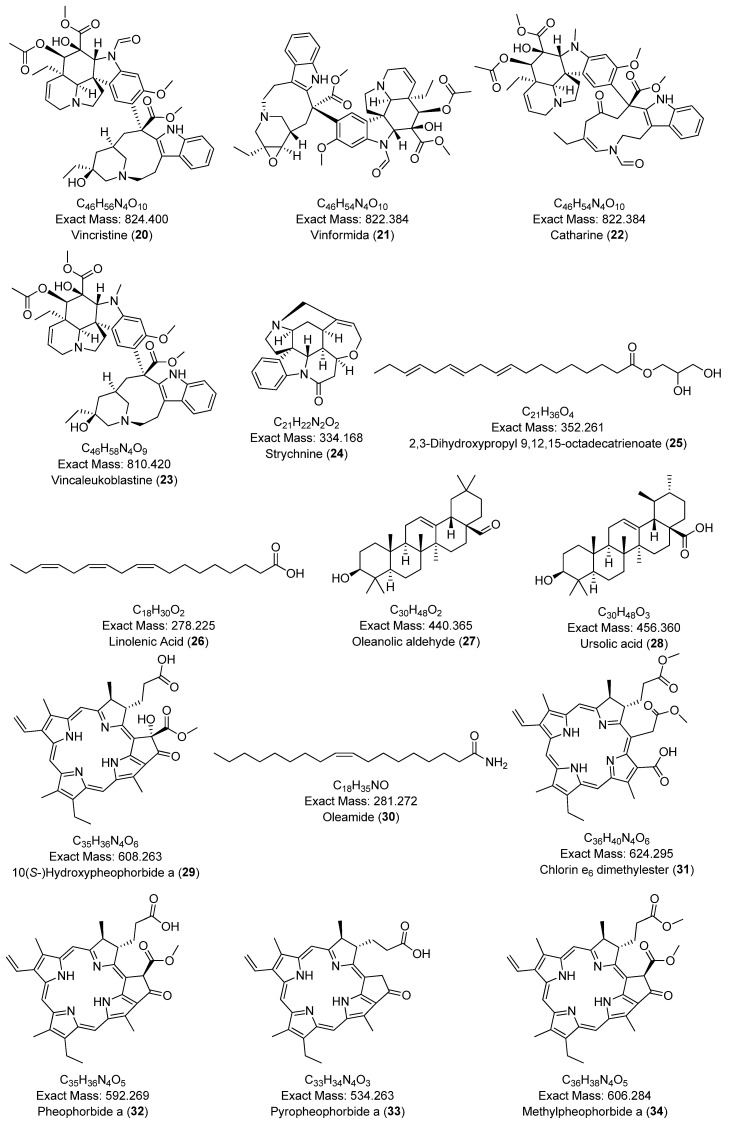
Chemical structures of annotated compounds from the methanolic leaf extract (A01) of *C. roseus*, identified by HPLC-ESI-MS/MS analysis.

A comprehensive phytochemical profiling of the methanolic leaf extract of *C. roseus* (A01) was conducted using HPLC-ESI-HRMS/MS, based on its antibacterial and free radical scavenging activities. Figure 3 presents the total ion chromatogram (TIC) with annotated metabolites. A total of 34 secondary metabolites—including 22 indole alkaloids, 2 fatty acids, 2 pentacyclic triterpenoids, 1 amino acid, 4 porphyrin derivatives, 1 glyceride, and 1 chlorin derivative—were identified in positive ionization mode, as listed in Table 2. To the best of our knowledge, two metabolites—2,3-dihydroxypropyl 9,12,15-octadecatrienoate and (10*S*)-hydroxypheophorbide A—were identified for the first time in *C. roseus*. A few peaks could not be annotated and are marked with an asterisk (*) due to low signal intensity and insufficient fragmentation peaks. The chemical structures of the annotated metabolites are illustrated in Figure 4. The BPCs and MS profiles of all detected metabolites are presented in Figures S7–S40.

### 2.6. GNPS Analysis

**Figure 5:**
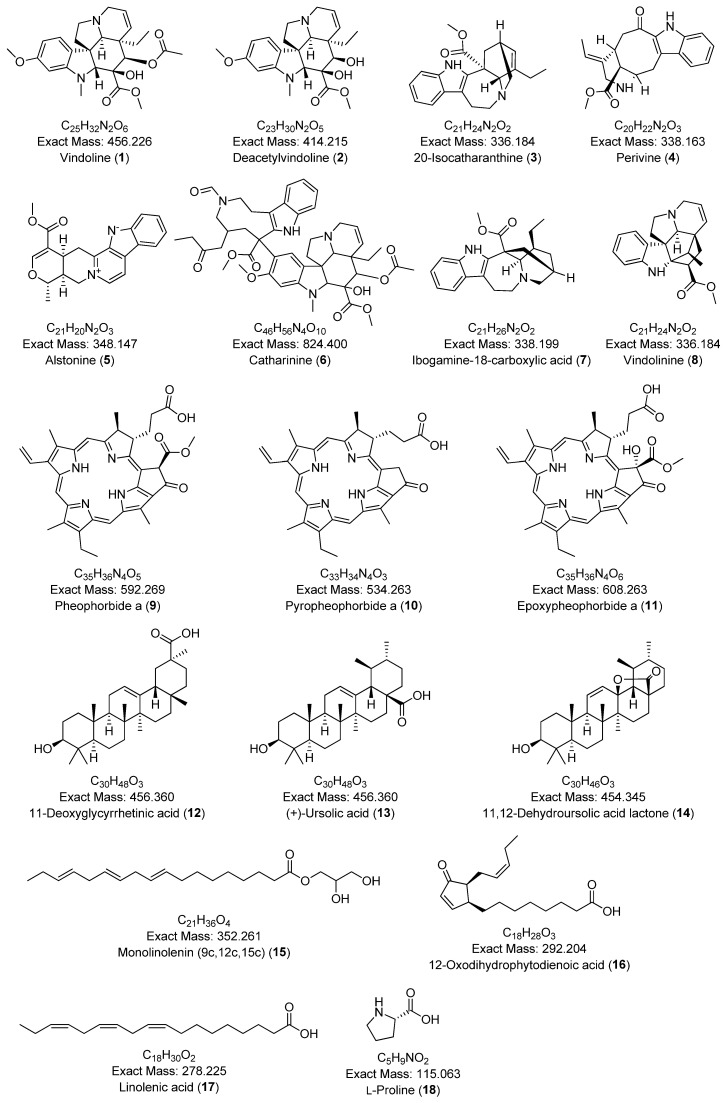
Chemical structures of annotated specialized metabolites from the methanolic leaf extract (A01) of *C. roseus* identified via GNPS analysis.

Given the radical scavenging activity and notable antimicrobial efficacy of the methanolic leaf extract of *C. roseus* (A01) against *Staphylococcus aureus* and *Shigella sonnei*, a comprehensive phytochemical profile was generated using MS/MS analysis and a GNPS-based metabolomics platform. A total of 18 compounds were tentatively identified from the leaf extract (A01), including 11 tryptophan-derived alkaloids, 3 triterpenoids, 3 fatty acids, and 1 amino acid, as listed in Table 3. The chemical structures of these compounds are shown in Figure 5.

**Figure 6:**
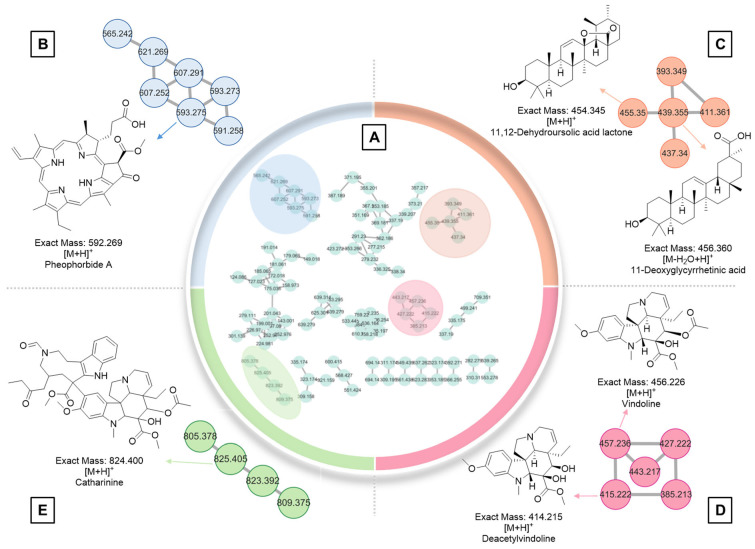
GNPS analysis and annotated specialized metabolites from the methanolic leaf extract (A01) of *C. roseus*: (**A**) Overview of the GNPS and classification of annotated compounds. (**B**) Enlarged view of the cluster containing pheophorbide A. (**C**) Enlarged view of the triterpenoid cluster. (**D**) Enlarged view of the tryptophan alkaloid cluster. (**E**) Enlarged view of the catharanthine cluster.

Molecular networking is a computational tool that visually represents the chemical space of complex samples. It operates on the principle that structurally similar molecules tend to produce comparable fragmentation patterns in tandem mass spectrometry (MS/MS). Each metabolite is represented as a node, typically labeled with its *m*/*z* (mass-to-charge) value, and nodes are grouped into clusters based on similarities in their MS/MS fragmentation profiles. In the methanolic leaf extract of *C. roseus* (A01), 176 molecular ions were detected and connected by 219 edges, forming a network. These ions were distributed into 10 multi-node clusters, one cluster with three nodes, eight clusters with two nodes, and 75 single, unconnected nodes (Figure 6A). Among these, four major clusters were identified (Figure 6). One node at *m*/*z* 593.275 was annotated as pheophorbide a (Figure 6B). Two ions at *m*/*z* 455.350 and 456.360 corresponded to the triterpenoids, 11,12-dehydroursolic acid lactone and 11-deoxoglycyrrhetinic acid, respectively (Figure 6C). Previous work by Huang et al. demonstrated the triterpene biosynthetic capacity of *C. roseus* through the identification of two key enzymes, CrAS and CrAO, which are essential for the formation of pentacyclic triterpenes such as ursolic acid and oleanolic acid [52]. In addition, vindoline—one of the most abundant metabolites in *C. roseus*—was detected at *m*/*z* 457.236 (Figure 6D). Deacetylvindoline (*m*/*z* 415.222) was also found in the same cluster, suggesting a potential biochemical relationship or precursor-product linkage. Another metabolite, catharanthine, an indole alkaloid with a molecular ion at *m*/*z* 825.405, was identified as one of more than 130 known terpenoid indole alkaloids in *C. roseus* (Figure 6E) [16].

**Table 3:** Annotated secondary metabolites from the methanolic leaf extract (A01) of *C. roseus* identified through GNPS analysis.

No	Annotated Compound	Accurate Mass (Da)	Precursor Ion	Adduct Type	MS^2^ Fragmentation Pattern	Molecular Formula	Retention Time (mins)	Error (ppm)	Reference
**Tryptophan alkaloids**									
**1**	Vindoline	456.226	457.230	[M+H]^+^	188.106	C_25_H_32_N_2_O_6_	9.24	13.1	[53]
**2**	Deacetylvindoline	414.215	415.220	[M+H]^+^	188.101, 173.078	C_23_H_30_N_2_O_5_	8.07	4.8	[54]
**3**	20-Isocatharanthine	336.184	337.191	[M+H]^+^	144.081, 93.070	C_21_H_24_N_2_O_2_	8.54	3.0	[55]
**4**	Perivine	338.163	339.17	[M+H]^+^	234.127, 144.080, 130.065, 93.069	C_20_H_22_N_2_O_3_	6.18	3.0	[56]
**5**	Alstonine	348.147	349.155	[M+H]^+^	235.087, 207.092	C_21_H_20_N_2_O_3_	8.65	2.9	[43]
**6**	Catharinine	824.400	825.407	[M+H]^+^	765.385, 556.280, 341.186, 144.081	C_46_H_56_N_4_O_10_	10.87	2.4	[57]
**7**	Ibogamine-18-carboxylic acid	338.199	339.207	[M+H]^+^	339.208, 144.081	C_21_H_26_N_2_O_2_	8.66	0.0	[58]
**8**	Vindolinine	336.184	337.19	[M+H]^+^	320.163, 177.090, 144.080, 117.069	C_21_H_24_N_2_O_2_	6.84	0.0	[37]
**9**	Pheophorbide a	592.269	593.269	[M+H]^+^	593.276, 533.255	C_35_H_36_N_4_O_5_	20.81	10.2	[50]
**10**	Pyropheophorbide a	534.263	535.270	[M+H]^+^	535.270, 507.275, 435.254	C_33_H_34_N_4_O_3_	21.07	1.9	[50]
**11**	Epoxypheophorbide a	608.263	609.272	[M+H]^+^	609.272, 591.261, 559.235, 531.240	C_35_H_36_N_4_O_6_	19.99	1.6	[50]
**Triterpenoids**									
**12**	11-Deoxyglycyrrhetinic acid	456.360	439.357	[M-H_2_O+H]^+^	439.357, 189.164, 121.101, 95.086	C_30_H_48_O_3_	7.32	4.5	[59]
**13**	(+)-Ursolic acid	456.360	457.368	[M+H]^+^	189.163, 95.085	C_30_H_48_O_3_	19.31	4.4	[60]
**14**	11,12-Dehydroursolic acid lactone	454.345	455.352	[M+H]^+^	437.344, 247.170, 133.101, 119.086	C_30_H_46_O_3_	18.31	4.3	[61]
**Fatty acids**									
**15**	Monolinolenin (9c,12c,15c)	352.261	353.270	[M+H]^+^	261.221, 95.086, 81.070, 67.055	C_21_H_36_O_4_	17.76	11.1	[62]
**16**	12-Oxodihydrophytodienoic acid	292.204	277.216	[M-H_2_O+H]^+^	235.170, 107.087, 93.071, 79.056	C_18_H_28_O_3_	14.84	3.6	[63]
**17**	Linolenic acid	278.225	279.232	[M+H]^+^	95.085, 81.069 67.054	C_18_H_30_O_2_	17.24	0.0	[64]
**Amino acid**									
**18**	L-Proline	115.063	116.071	[M+H]^+^	116.071, 70.065	C_5_H_9_NO_2_	0.99	17.2	[65]

## 3. Discussion

*Catharanthus roseus* is rich in organic bioactive compounds, primarily alkaloids, flavonoids, and polyphenolic compounds, which feature diverse functional groups such as hydroxyl, carbonyl, and heterocycles [65]. Given its significant ethnobotanical value and wide-ranging applications, further untargeted metabolomic profiling is essential to uncover additional secondary metabolites and to validate its broader therapeutic potential. This study primarily aimed to assess the antioxidant, antimicrobial, and cytotoxic activities of *C. roseus* extracts, followed by the annotation of secondary metabolites to confirm and expand upon previous experimental findings.

The radical scavenging activity of the methanolic leaf extract of *C. roseus* (A01) was found to be 53.12 ± 1.60 µg/mL, closely aligning with a previously reported value of 49.74 ± 0.52 µg/mL [28]. This activity may be attributed to the presence of alkaloids and flavonoids, which are known for their free radical-scavenging properties and ability to reduce oxidative stress. Additionally, metabolites such as quercetin, ursolic acid, vindoline, coronaridine, alstonine, ajmalicine, and coronaridine have been reported to contribute to the moderate radical scavenging potential of *C. roseus* [66,67,68,69]. The extract also demonstrated weak antibacterial activity against *Shigella sonnei*, *Staphylococcus aureus*, and *Klebsiella pneumoniae*, but showed no efficacy against *Escherichia coli*. Furthermore, the brine shrimp lethality assay of A01 revealed cytotoxic potential, with an LC_50_ value of less than 1000 µg/mL. Although a correlation was observed between radical scavenging activity and lethality, this does not imply a causal relationship. The methanolic extract (A01) also exhibited significant cytotoxicity against HeLa and A549 cell lines, with IC_50_ values of 37.5 ± 0.10 µg/mL and 35.98 ± 0.89 µg/mL, respectively. Further studies are required to isolate the specific bioactive constituents responsible for these effects and to elucidate their underlying mechanisms of action. Alkaloids such as coronaridine, vindorosine, vincristine, vinformide, catharanthine, alstonine, and porphyrin derivatives have previously been reported to possess cytotoxic activity, supporting the findings from the MTT and brine shrimp assays [16,65,69].

Based on the effective biological activities observed in the methanolic leaf extract of *C. roseus*, metabolic profiling was performed using HPLC-ESI-HRMS/MS analysis, resulting in the identification of 34 secondary metabolites. Most of these were indole alkaloids, including terpene indole alkaloids, corynanthean-type, tryptophan-derived, oxindole, and *β*-carboline alkaloids. L-proline, an amino acid known to accumulate under saline stress conditions, was also detected [70]. Preakuammicine, a biosynthetic intermediate exclusively detected in *C. roseus*, has not yet been isolated but is considered crucial in the formation of type II and type III monoterpene indole alkaloids, such as strychnine, ajmalicine, and serpentine [28,71]. Quercetin, a well-known flavonoid with antioxidant and anti-inflammatory properties, is associated with cardiovascular and neuroprotective effects [67]. Perivine, previously reported in both *Lanceus pich* and *C. roseus*, exhibits potent extracellular virucidal activity against the Vaccinia virus, the causative agent of smallpox [29]. Mitraphylline, identified in *Uncaria tomentosa* and *C. roseus*, is known to inhibit cytokine production and suppress tumor necrosis factor-α (TNF-α) expression, contributing to its anti-inflammatory potential [30,72]. Several alkaloids—including catharanthine, tabersonine, and vindolinine—share the same molecular mass and are closely linked within the indole alkaloid biosynthetic pathway. Catharanthine serves as a key precursor to tabersonine [73], while vindoline can be derived from either catharanthine or tabersonine through enzymatic transformations such as oxidation, methylation, and reduction [74]. Vindoline and catharanthine are further coupled through peroxidase-mediated reactions to form vinblastine [75]. Pleiocarpamine, derived from geissoschizine, has been reported to exhibit neuroprotective effects and is used in the treatment of neurological disorders [76]. Deacetylvindoline, an essential intermediate in vindoline biosynthesis, is produced via hydroxylation of desacetoxyvindoline [77]. Vindoline, in turn, is a crucial precursor for both vinblastine and vincristine [38], and exhibits various pharmacological activities including antioxidant, antihyperlipidemic, antiproliferative, and antidiabetic effects [68]. Understanding the biosynthetic pathways leading to vinblastine and vincristine is vital for pharmaceutical development. Additionally, tubotaiwine, first identified in *Pleiocarpa tubicina*, has shown strong antiplasmodial activity, with an IC_50_ value of 8.5 Μm [38,78]. Coronaridine, found in *Tabernaemontana catharinensis* and related species, has demonstrated cytotoxic activity by inducing cell death in hepatocellular carcinoma cells, though it caused minimal DNA damage and had limited effects on the plasma membrane [79]. Similarly, alstonine, a tryptophan-derived alkaloid, has been shown to possess anxiolytic, antipsychotic, antiproliferative, and antimalarial activities [69]. Moreover, previous studies have shown that ajmalicine, found in *Rauvolfia*, *Mitragyna* species, and *C. roseus*, exhibits antimicrobial, antihypertensive, and vasodilatory properties [80]. Similarly, vindorosine, vincristine, vinformide, and catharanthine have demonstrated anticancer activity by inducing apoptosis and disrupting microtubule dynamics, thereby inhibiting cell division [16]. In the present study, strychnine—previously reported in *Strychnos* species—was also identified. This compound is a potent convulsant that enhances neuronal activity in the central nervous system by inhibiting glycine receptors [81]. Strychnine is biosynthesized through a sequence of oxidation and reduction reactions from geissoschizine to Wieland–Gumlich aldehyde [82]. Additionally, 2,3-dihydroxypropyl 9,12,15-octadecatrienoate—previously isolated from *Citrullus colocynthis* and *Chenopodium album*—has been reported to exhibit central nervous system depressant, anti-inflammatory, and antifungal activities [83,84]. To the best of our knowledge, this is the first report of its detection in *C. roseus*. Two fatty acids were also annotated in this study. Linolenic acid, commonly found in *Glycine max*, is known for its anti-inflammatory and antioxidant effects and has therapeutic potential for cardiovascular and neurological diseases [85]. Oleamide, another fatty acid identified, is known to induce sleep, stimulate vanilloid receptors, and mimic neurotransmitter activity at cannabinoid receptors, suggesting a potential role in cardiac muscle development [86]. Furthermore, two pentacyclic triterpenoids—ursolic acid and oleanoic aldehyde—were identified. Ursolic acid is known for its antimicrobial, antiviral, antioxidant, and hepatoprotective properties, while oleanoic aldehyde, typically found in grapes, has been reported to promote insulin synthesis and reduce blood glucose levels, functioning as an antihyperglycemic agent [87]. In addition, four porphyrin derivatives were annotated, all of which are known as photodynamic sensitizers with demonstrated anticancer potential [88].

The GNPS technique enabled the simultaneous analysis of multiple mass spectra, allowing for enhanced visualization and interpretation of complex spectral datasets [89]. This approach also revealed structural relationships among molecules, resulting in the identification of 18 metabolites. However, many polyphenols and flavonoids were not detected, likely due to matrix effects or ion suppression. Ion suppression occurs when co-eluting compounds in a sample matrix interfere with the ionization of target analytes, thereby reducing signal intensity or preventing detection [90]. Several metabolites identified in this study—such as perivine, vindolinine, catharanthine, vindoline, ajmalicine, ursolic acid, and vincristine—have been previously reported to exhibit antimicrobial activity, which supports the antimicrobial effects observed in the methanolic leaf extract [91,92]. Metabolic profiling not only enhances our understanding of biosynthetic pathways but also provides opportunities to identify and manipulate key metabolic steps for the generation of novel bioactive compounds with therapeutic potential. Future studies should focus on isolating individual metabolites, followed by detailed structural elucidation using techniques such as NMR spectroscopy and comprehensive bioactivity evaluations to assess their pharmaceutical relevance. Furthermore, optimization of extraction protocols may enhance metabolite recovery and facilitate the discovery of previously undetectable compounds, thereby contributing to the advancement of pharmacological research.

## 4. Materials and Methods

### 4.1. Chemicals

Solvents, including methanol (≥99.0%), ethanol (≥99.9%), hexane (≥99.0%), di-chloromethane (DCM, ≥99.0%), and ethyl acetate (≥99.5%), were of analytical grade from Merck (Darmstadt, Germany) and Thermo Fischer Scientific (Mumbai, India). Gallic acid (≥98.0%), potassium acetate (≥99.0%), and Folin–Ciocalteu’s (FC) phenol reagent were purchased from Loba Chemie (Mumbai, India). In addition, anhydrous sodium carbonate (≥98.5%), tannic acid (≥99.5%), iron(III) nitrate nonahydrate (≥98.0%), Mueller–Hinton broth, Mueller–Hinton agar, and Mueller agar growth nutrients were purchased from HiMedia Laboratories (Mumbai, India). Similarly, dimethyl sulfoxide (DMSO, ≥99.0%) was purchased from the Silico research laboratory (Madhya Pradesh, India). Acetic acid (≥99.7%) was obtained from Control Drug House (Gujarat, India). Quercetin (≥95.0%) and 2,2-diphenyl-1-picrylhydrazyl (DPPH, ≥95.0%) were procured from Srikem Laboratories (Mumbai, India). Positive controls, neomycin and doxorubicin (≥98.0%), were purchased from Sigma-Aldrich (St. Louis, MO, USA).

### 4.2. Plant Collection and Extract Preparation

*Catharanthus roseus* (L.) G. Don (stems and leaves) was collected in July 2023 from the Kathmandu Valley, Nepal (27°42′2.7684″ N, 85°18′0.5040″ E), based on its traditional medicinal use. The species was identified at the National Herbarium and Plant Laboratories, Godawari, Nepal, and registered under voucher number RB01. The collected plant materials were air-dried, crushed, powdered, and stored in airtight plastic bags for further processing. Plant extracts were prepared using the maceration technique in four different solvents: methanol, ethyl acetate, dichloromethane (DCM), and hexane, as detailed in the Supplementary Materials. Leaf extracts were labeled as A01 (methanol), A02 (ethyl acetate), A03 (DCM), and A04 (hexane), while stem extracts were designated A05 (methanol) and A06 (ethyl acetate).

### 4.3. DPPH Radical Scavenging Assay

DPPH is a stable free radical that exhibits a strong absorbance at 517 nm, which decreases upon reduction by an antioxidant. The radical scavenging assay was performed according to the protocol described by Blois (1958) [93]. Briefly, 100 µL of each extract at various concentrations was dispensed in triplicate into 96-well plates. For the negative control, 100 µL of methanol (for samples A01 and A05) or 50% DMSO (for A02, A03, A04, and A06) was used, depending on the solvent used for extract dissolution. Initial absorbance was measured at 517 nm using a microplate spectrophotometer, followed by the addition of 100 µL of 0.1 mM DPPH solution to each well. The plates were incubated in the dark for 30 min, after which the final absorbance was recorded. Quercetin was used as the positive control. The degree of color change from purple to yellow indicated scavenging activity, and the percentage of radical scavenging was calculated using the following equation [94]:

$$\%Inhibition=\frac{A_{control}-A_{extract}}{A_{control}}\times 100$$

A_control_ = Absorbance of DPPH solution (control, without sample);

A_extract_ = Absorbance of the test sample or reference sample and DPPH.

GraphPad Prism 8.0.2 (accessed on 4 January 2025) was used to calculate the IC_50_ value of different extracts of *C. roseus* for DPPH radical scavenging activity. All data are presented as mean ± standard deviation (n = 3). A two-way ANOVA with replication was performed, followed by Tukey’s HSD post hoc test to assess the effects of extract type and concentration on the DPPH assay. A *p*-value < 0.05 was considered statistically significant.

### 4.4. Antimicrobial Assay

The antimicrobial activity of the extracts was evaluated using the agar well diffusion method on Mueller–Hinton Agar (MHA) plates [95]. Minimum inhibitory concentration (MIC) and minimum bactericidal concentration (MBC) were determined using the broth microdilution method, following the guidelines of the Clinical and Laboratory Standards Institute (CLSI) [96], as detailed in the Supplementary Materials. The antimicrobial activity of various extracts of *C. roseus* was determined against ATCC strains, including gram-positive bacteria, *Staphylococcus aureus* ATCC 43300, and Gram-negative bacteria, *Escherichia coli* ATCC 25912, *Klebsiella pneumoniae* ATCC 700603, and *Shigella sonnei* ATCC 25931.

### 4.5. Brine Shrimp Assay

The brine shrimp lethality assay is widely used to evaluate the toxicity and bioactivity of physiologically active compounds, including those from plant extracts. In this study, the assay was conducted using methanol-based leaf (A01) and stem (A05) extracts of *Catharanthus roseus*, following the standard protocol described by Olowa and Nuñeza (2013) [97]. To prepare the stock solution, 20 mg of the methanolic leaf extract (A01) was dissolved in 2 mL of 50% DMSO to yield a concentration of 10,000 ppm. Serial dilutions were performed to obtain seven concentrations: 1000, 800, 500, 250, 125, 100, and 50 µg/mL. For each concentration, 500 µL of the solution was added to separate test tubes containing 4 mL of artificial seawater and 10 matured brine shrimp nauplii. Each concentration was tested in triplicate (21 test tubes in total). After 24 h of incubation, the number of surviving nauplii was recorded using disposable pipettes. Potassium dichromate served as the positive control, while 50% DMSO was used as the negative control. For the assay, the brine shrimp (*Artemia salina*) cysts (100 mg; Oji Art Industries, Soka city, Japan) were hatched in 1 L of artificial seawater prepared by dissolving the required salts in distilled water (see Table S4 for composition). The hatching chamber was maintained at 30 °C under continuous illumination (100 W white lamp, ~2000 lux) with gentle aeration. After 48 h, the resulting 48-h-old nauplii, which aggregated on the illuminated side, were collected for the lethality assay.

### 4.6. Cytotoxicity Assay

Cytotoxicity was assessed using both the MTT assay and the RealTime-Glo™ MT Cell Viability Assay on HeLa (cervical cancer) and A549 (lung carcinoma) cell lines. For subculturing, Dulbecco’s Modified Eagle Medium (DMEM; Sigma-Aldrich) supplemented with penicillin–streptomycin and fetal bovine serum (FBS) was used to prepare the complete growth medium. Cells were seeded in 96-well plates at a density of approximately 1 × 10^5^ to 1 × 10^6^ cells per well in 100 µL of growth medium. Cell numbers were determined using a hemocytometer. The plates were incubated overnight in a CO_2_ incubator at 37 °C with 5% CO_2_ and 95% humidity. After incubation, 100 µL of *C. roseus* methanolic extract (from both leaves and stems), prepared at various concentrations in 1% DMSO, was added to each well. After 48 h of treatment, the medium was replaced with fresh medium, and 20 µL of MTT stock solution (5 mg/mL) was added to each well. The plate was incubated for an additional 4 h in the CO_2_ incubator. Following incubation, the supernatant was removed, and 100 µL of DMSO was added to each well to dissolve the formazan crystals. After 30 min of gentle shaking at room temperature, absorbance was measured at 570 nm using a microplate reader (Synergy LX Multimode Reader, BioTek, Winooski, VT, USA) [98]. GraphPad Prism 8.0.2 (accessed from 8 February 2025) was used to calculate the IC_50_ value of different extracts of *C. roseus* for the cytotoxicity assay.

### 4.7. Real-Time-Glo™ MT Cell Viability Assay

The RealTime-Glo™ MT Cell Viability Assay was used to determine the number of viable cells based on luminescence, which reflects the metabolic activity of live cells. The assay was performed according to the standard protocol provided in the Promega Technical Bulletin. HeLa cells were seeded at appropriate densities into 96-well plates. Serially diluted methanolic extracts from both the stem and leaf of *C. roseus* were added to the wells and incubated for 24 h. Doxorubicin was used as the positive control, and 1% DMSO served as the negative control. To prepare the 2× RealTime-Glo reagent, 996 µL of culture medium, 1 mL of RealTime-Glo buffer, and 2 µL each of NanoLuc^®^ enzyme and MT cell viability substrate (both at 1000× concentration) were combined and mixed thoroughly using a vortex mixer. Equal volumes of the prepared reagent were then added to each well. The plate was incubated for 1 h, after which luminescence was measured using a microplate luminometer (Synergy LX Multimode Reader, BioTek, Winooski, VT, USA) with an integration time of 0.25 s per well.

### 4.8. Untargeted Metabolomics Using HPLC-ESI-HRMS/MS Analysis

The methanolic leaf extract of *C. roseus* (A01) was chemically profiled using high-performance liquid chromatography–electrospray ionization–high-resolution tandem mass spectrometry (HPLC-ESI-HRMS/MS) on an Agilent G6545B quadrupole time-of-flight (Q-TOF) mass spectrometer (Agilent Technologies, Santa Clara, CA, USA) at Sungkyunkwan University. The sample was prepared at a concentration of 2 mg/mL in HPLC-grade solvent and injected into the autosampler in 150 µL aliquots. Chromatographic separation was achieved using an Acquity^®^ UPLC BEH reverse-phase C18 column (150 mm × 2.1 mm, 1.7 µm particle size; Waters Corporation, Milford, MA, USA). The mobile phases consisted of water (A) and acetonitrile (B), both acidified with 0.1% formic acid. The gradient program was as follows: 5% B (0–2 min), 20% B (5 min), 100% B (20 min), and returning to 5% B (23–25 min). The flow rate was maintained at 0.4 mL/min with an injection volume of 3 µL. Mass spectrometric data were acquired in positive ion mode over an *m*/*z* range of 50–1700 Da, with a full width at half maximum (FWHM) of 3000. Collision energies were set at 15 V and 40 V for MS/MS fragmentation. Mass spectral data were acquired and processed using Agilent MassHunter Workstation Software LC/MS Data Acquisition for 6200 series TOF/6500 series Q-TOF, version B.09.00, build 9.0.9044.1 SP1 (Agilent Technologies, Santa Clara, CA, USA).

### 4.9. Metabolic Profiling and Identification of Secondary Metabolites

Secondary metabolites were identified and annotated using MestReNova software (version 12.0.0; https://mestrelab.com/; accessed 6–30 March 2024) [99]. Molecular ions were determined based on the base peak chromatogram (BPC). Precursor ions were primarily detected in the protonated form [M+H]^+^, with some observed as sodium adducts [M+Na]^+^, [2M+H]^+^, and [2M+Na]^+^ in positive ion mode. Annotation of molecular ion peaks with strong and distinct fragment ions was performed using public databases, including PubChem (https://pubchem.ncbi.nlm.nih.gov/; accessed 25–30 March 2024) [100], ChemSpider (http://www.chemspider.com/; accessed 25–30 March 2024) [101], and LOTUS (https://lotus.naturalproducts.net/; accessed 25–30 March 2024) [102]. The literature references were also consulted for further confirmation. Additionally, the raw data were converted into Mascot Generic Format (.mgf) files using the ProteoWizard tool [103]. The resulting .mgf files were analyzed using SIRIUS (version 5.8.0; accessed 7–30 March 2024) for molecular formula prediction, compound validation, and dereplication of unknown metabolites based on MS^2^ fragmentation patterns.

### 4.10. Molecular Networking with GNPS

Raw spectral files in “.d” format were first converted to “.mzXML” format and uploaded to the GNPS platform via the recommended FTP client. Molecular networking was performed using established GNPS workflows_ for MS/MS data analysis and visualization. The resulting molecular network was exported in “.graphml” format and imported into Cytoscape (version 3.9.1) for further analysis and customized visualization.

## 5. Conclusions

This study comprehensively evaluated the antioxidant, antimicrobial, and cytotoxic properties of *C. roseus* leaf and stem extracts, with a particular focus on the methanolic leaf extract (A01). The leaf extract demonstrated mild DPPH radical scavenging activity and weak antibacterial efficacy, particularly against *Staphylococcus aureus* and *Shigella sonnei*. Cytotoxic assays revealed moderate activity against HeLa and A549 cancer cell lines, with IC_50_ values supporting moderate anticancer potential. Untargeted metabolomic profiling using HPLC-ESI-HRMS/MS led to the annotation of 34 secondary metabolites, including indole alkaloids, triterpenoids, fatty acids, and porphyrin derivatives. Notably, several bioactive compounds such as vindoline, catharanthine, vincristine, and ursolic acid—previously associated with antioxidant, antimicrobial, and antiproliferative effects—were identified. Two metabolites, 2,3-dihydroxypropyl 9,12,15-octadecatrienoate and (10*S*)-hydroxypheophorbide A, were reported in *C. roseus* for the first time. GNPS analysis further corroborated the metabolite diversity and revealed structural relationships within key compound classes such as tryptophan-derived alkaloids and triterpenes. Taken together, these findings highlight the metabolic richness and therapeutic potential of *C. roseus*, particularly its leaves. The integration of biological assays with advanced metabolomics offers valuable insight into the plant’s bioactive constituents. Future investigations should focus on the isolation of individual metabolites, detailed structural characterization, and mechanistic studies to further explore their pharmacological applications.

## Figures and Tables

**Table 1:** DPPH radical scavenging potential of the leaf extracts of *C. roseus* and quercetin as a positive control.

Extracts ^1^	IC_50_ (µg/mL)
A01	53.12 ± 1.60
A02	94.12 ± 1.03
A03	92.69 ± 0.90
A04	54.27 ± 1.48
Quercetin ^2^	3.894 ± 0.68

^1^ A01, A02, A03, and A04 represent the methanolic, ethyl acetate, DCM, and hexane extracts of *C. roseus* leaves, respectively. ^2^ positive control. All data are presented as mean ± standard deviation (n = 3).

**Table 2:** Annotated secondary metabolites identified in positive ion mode by HPLC-ESI-MS/MS analysis of the methanolic leaf extract (A01) of *C. roseus*.

No	Annotated Compound	Exact Mass	Observed Mass	Detected Ion	Molecular Formula	RDBE ^1^	Absolute Error (ppm)	Retention Time (min)	CSI Finger ID Score (%)	References
**1**	L-Proline	115.062	116.071	[M+H]^+^	C_5_H_9_NO_2_	2.0	0.82	1.00	-	[6]
**2**	Preakuammicine	352.178	353.186	[M+H]^+^	C_21_H_24_N_2_O_3_	11.0	0.11	5.80	70.44	[28]
**3**	Quercetin	302.042	303.049	[M+H]^+^	C_15_H_10_O_7_	11.0	2.18	6.03	-	[19]
**4**	Perivine	338.162	339.17	[M+H]^+^	C_20_H_22_N_2_O_3_	11.0	2.29	6.27	71.65	[29]
**5**	Mitraphylline (Ajmalicine oxindole B)	368.173	369.181	[M+H]^+^	C_21_H_24_N_2_O_4_	11.0	1.05	6.44	68.86	[30]
**6**	Catharanthin	336.400	337.192	[M+H]^+^	C_21_H_24_N_2_O_2_	11.0	3.32	6.71	92.76	[31]
**7**	Tabersonine	336.180	337.192	[M+H]^+^	C_21_H_24_N_2_O_2_	11.0	0.19	7.07	63.10	[30]
**8**	Yohimbine	354.193	355.200	[M+H]^+^	C_21_H_26_N_2_O_3_	10.0	1.03	7.25	93.60	[32]
**9**	Geissoschizine	352.178	353.186	[M+H]^+^	C_21_H_24_N_2_O_3_	11.0	0.77	7.39	81.20	[33]
**10**	Quebrachidine (Vincarine)	352.178	353.186	[M+H]^+^	C_21_H_24_N_2_O_3_	11.0	1.11	7.52	77.30	[34]
**11**	Pleiocarpamine	322.167	323.176	[M+H]^+^	C_20_H_22_N_2_O_2_	11.0	0.60	7.80	-	[35]
**12**	Deacetylvindoline	414.214	415.223	[M+H]^+^	C_23_H_30_N_2_O_5_	10.0	0.22	8.07	-	[36]
**13**	Vindolinine	336.180	337.192	[M+H]^+^	C_21_H_24_N_2_O_2_	11.0	1.76	8.16	59.86	[37]
**14**	Tubotaiwine	324.183	325.191	[M+H]^+^	C_20_H_24_N_2_O_2_	10.0	0.08	8.34	97.62	[38]
**15**	Alstonine	348.147	349.155	[M+H]^+^	C_21_H_20_N_2_O_3_	13.0	1.72	8.43	78.59	[39]
**16**	Coronaridine	338.200	339.207	[M+H]^+^	C_21_H_26_N_2_O_2_	10.0	1.57	8.61	85.46	[40]
**17**	Ajmalicine	352.178	353.186	[M+H]^+^	C_21_H_24_N_2_O_3_	11.0	0.46	8.75	86.01	[33]
**18**	Vindoline	456.225	457.235	[M+H]^+^	C_25_H_32_N_2_O_6_	11.0	2.94	9.20	97.78	[41]
**19**	Vindorosine	426.214	427.222	[M+H]^+^	C_24_H_30_N_2_O_5_	11.0	1.32	9.25	-	[42]
**20**	Vincristine	824.400	825.407	[M+H]^+^	C_46_H_56_N_4_O_10_	21.0	0.10	10.88	94.99	[43]
**21**	Vinformida	822.383	823.392	[M+H]^+^	C_46_H_54_N_4_O_10_	22.0	2.51	11.06	92.29	[44]
**22**	Catharine	822.383	823.392	[M+H]^+^	C_46_H_54_N_4_O_10_	22.0	0.87	11.47	85.52	[44]
**23**	Vincaleukoblastine	822.383	823.392	[M+H]^+^	C_46_H_54_N_4_O_10_	22.0	0.87	11.83	88.57	[44]
**24**	Strychnine	334.167	335.176	[M+H]^+^	C_21_H_22_N_2_O_2_	12.0	0.96	14.55	-	[45]
**25**	2,3-Dihydroxypropyl 9,12,15-octadecatrienoate	352.260	353.268	[M+H]^+^	C_21_H_36_O_4_	4.0	0.60	17.77	95.83	[46]
**26**	Linolenic acid	278.224	279.232	[M+H]^+^	C_18_H_30_O_2_	4.0	1.56	18.72	99.27	[46]
**27**	Oleanolic aldehyde	438.349	439.356	[M+H]^+^	C_30_H_46_O_2_	8.0	1.35	18.94	68.87	[47]
**28**	Ursolic acid	456.359	457.367	[M+H]^+^	C_30_H_48_O_3_	7.0	1.76	18.99	-	[47]
**29**	(10*S*)-Hydroxypheophorbide a	608.262	609.270	[M+H]^+^	C_35_H_36_N_4_O_6_	20.0	1.05	19.58	-	[48]
**30**	Oleamide	281.271	282.280	[M+H]^+^	C_18_H_35_NO	2.0	1.95	19.67	100	[46]
**31**	Chlorin e_6_ dimethylester	624.294	625.302	[M+H]^+^	C_36_H_40_N_4_O_6_	19.0	0.44	19.76	86.30	[49]
**32**	Pheophorbide a	592.268	593.276	[M+H]^+^	C_35_H_36_N_4_O_5_	20.0	0.44	20.35	89.80	[50]
**33**	Pyropheophorbide a	534.260	535.270	[M+H]^+^	C_33_H_34_N_4_O_3_	19.0	0.37	21.07	80.36	[50]
**34**	Methylpheophorbide a	606.283	607.294	[M+H]^+^	C_36_H_38_N_4_O_5_	20.0	0.28	21.62	83.07	[51]

^1^ Ring Double Bond Equivalents (RDBE) value represents the number of rings and/or double bonds present in a molecule.

## Data Availability

Data available on request from the authors.

## Supplementary Materials

The following supporting information can be downloaded at: https://www.mdpi.com/article/10.3390/plants14152395/s1, Table S1: Zone of inhibition of different extracts of *Catharanthus roseus*; Table S2: MIC and MBC of different extracts against two bacterial strains; Table S3: Calculation of LC_50_ of methanolic (leaves) (A01) extract of *C. roseus*; Table S4: Composition of artificial seawater; Table S5: Analysis of variance of DPPH radical scavenging activity; Table S6: post hoc Tukey HSD test of DPPH radical scavenging activity; Figure S1: Plot of % inhibition of A05 (methanolic extract of the stem) vs. concentration (µg/mL); Figure S2: Plot of % inhibition of A06 (ethyl acetate extract of the stem) vs. concentration (µg/mL); Figure S3: Antimicrobial activity of various extracts of *C. roseus* against *Shigella sonnei* and *Klebsiella pneumonia*; Figure S4: MIC of different extracts of *C. roseus* against *S. sonnei*; Figure S5: MIC of different extracts of *C. roseu* against *S. aureus* (For PC, dilution was started from 0.25 mg); Figure S6: MBC of different extract of leaves and stem of *C. roseus* along with positive control against *S. aureus* in the right and *S. sonnei* in the left; Figure S7: BPC and MS profile of L-proline (**1**); Figure S8: BPC and MS profile of preakuammicine (**2**); Figure S9: BPC and MS profile of quercetin (**3**); Figure S10: BPC and MS profile of perivine (**4**); Figure S11: BPC and MS profile of mitraphylline (ajmalicine oxindole B) (**5**); Figure S12: BPC and MS profile of catharanthin (**6**); Figure S13: BPC and MS profile of tabersonine (**7**); Figure S14: BPC and MS profile of yohimbine (**8**); Figure S15: BPC and MS profile of geissoschizine (**9**); Figure S16: BPC and MS profile of quebrachidine (vincarine) (**10**); Figure S17: BPC and MS profile of pleiocarpamine (**11**); Figure S18: BPC and MS profile of deacetylvindoline (**12**); Figure S19: BPC and MS profile of vindolinine (**13**); Figure S20: BPC and MS profile of tubotaiwine (**14**); Figure S21: BPC and MS profile of alstonine (**15**); Figure S22: BPC and MS profile of coronaridine (**16**); Figure S23: BPC and MS profile of ajmalicine (**17**); Figure S24: BPC and MS profile of vindoline (**18**); Figure S25: BPC and MS profile of vindorosine (**19**); Figure S26: BPC and MS profile of vincristine (**20**); Figure S27: BPC and MS profile of vinformida (formyl leurosine) (**21**); Figure S28: BPC and MS profile of catharine (**22**); Figure S29: BPC and MS profile of vincaleukoblastine (**23**); Figure S30: BPC and MS profile of strychnine (**24**); Figure S31: BPC and MS profile of 2,3-dihydroxypropyl 9,12,15-octadecatrienoate (**25**); Figure S32: BPC and MS profile of linolenic acid (**26**); Figure S33: BPC and MS profile of oleanolic aldehyde (**27**); Figure S34: BPC and MS profile of ursolic acid (**28**); Figure S35: BPC and MS profile of 10(*S*)-hydroxypheophorbide a (**29**); Figure S36: BPC and MS profile of oleamide (**30**); Figure S37: BPC and MS profile of chlorin e6 dimethylester (**31**); Figure S38: BPC and MS profile of pheophorbide a (**32**); Figure S39: BPC and MS profile of pyropheophorbide a (**33**); Figure S40: BPC and MS profile of methylpheophorbide a (**34**); Figure S41: Observed MS/MS profiles of the protonated molecular ions at *m*/*z* 353.268 (a) and *m*/*z* 609.270 (b); Figure S42. Observed fragmentation pattern of 2,3-dihydroxypropyl 9,12,15-octadecatrienoate in (+)-ESI mode; Figure S43. Observed fragmentation pattern of (10*S*)-hydroxypheophorbide a in (+)-ESI mode; Figure S44. A graphical representation of luminescence vs. concentration of *C. roseus* (stem and leaves)
