# Supplementary material for: HPLC-ESI-HRMS/MS-Based Metabolite Profiling and Bioactivity Assessment of Catharanthus roseus
Source: Plants (Basel). 2025 Aug 2;14(15):2395. doi: 10.3390/plants14152395 (PMC12348932; doi:10.3390/plants14152395)
Supplement: Supplementary file 1 [file plants-14-02395-s001.zip › plants-3738023-supplementary.pdf]

HPLC-ESI-HRMS/MS-Based Metabolite Profiling and Bioactivity Assessment of *Catharanthus roseus*

Soniya Joshi [1,†], Chen Huo [2,†], Rabin Budhathoki [1], Anita Gurung [1], Salyan Bhattarai [3], Khaga Raj Sharma [1], Ki Hyun Kim [2,*] and Niranjan Parajuli [1,*,‡]

[1] Central Department of Chemistry, Tribhuvan University, Kirtipur, Kathmandu 44618, Nepal;
soniyajoshi157@gmail.com (S.J.); rabin.bc.992@gmail.com (R.B.);
anitagurung9855@gmail.com (A.G.);
khagarajsharma41@gmail.com (K.R.S.)

[2] School of Pharmacy, Sungkyunkwan University, Suwon 16419, Republic of Korea;
huochen_0213@163.com

[3] Paraza Pharma, Inc., 2525 Marie-Curie Avenue, Montreal, QC H4S 2E, Canada;
salyanbiotech@gmail.com

* Correspondence: khkim83@skku.edu (K.H.K.); nparajuli@ncat.edu (N.P.);
Tel.: +82-31-290-7700 (K.H.K.)

† These authors contributed equally to this study.

‡ Current address: Department of Chemical, Biological, and Bioengineering, North Carolina A&T State
University, Greensboro, NC 27411, USA.

Contents

Table S1: Zone of inhibition of different extracts of *Catharanthus roseus* 4

Table S2: MIC and MBC of different extracts against two bacterial strains 4

Table S3: Calculation of LC_{50} of methanolic (leaves) (A01) extract of *C. roseus* 4

Table S4: Composition of artificial seawater...5

Table S5: Analysis of variance of DPPH radical scavenging activity...5

Table S6: post-hoc Tukey HSD test of DPPH radical scavenging activity...5

Figure S1: Plot of % inhibition of A05 (Methanolic extract of the stem) vs Concentration (µg/mL) 6

Figure S2: Plot of % inhibition of A06 (Ethyl acetate extract of the stem) vs Concentration (µg/mL)....... 6

Figure S3: Antimicrobial activity of various extracts of *C. roseus* against *Shigella sonnei* and *Klebsiella pneumonia*... 7

Figure S4: MIC of different extracts of *C. roseus* against *S. sonnei* (For PC, dilution was started from 0.25mg).. 7

Figure S5: MIC of different extracts of *C. roseus* against *S. aureus* (For PC, dilution was started from 0.25mg).. 8

Figure S6: MBC of different extract of leaves and stem of *C. roseus* along with positive control against *S. aureus* in the right and *S. sonnei* in the left.. 10

Figure S7: BPC and MS profile of L-proline (1) 11

Figure S8: BPC and MS profile of preakuammicine (2)... 11

Figure S9: BPC and MS profile of quercetin (3) 12

Figure S10: BPC and MS profile of perivine (4)... 12

Figure S11: BPC and MS profile of mitraphylline (ajmalicine oxindole B) (5)... 13

Figure S12: BPC and MS profile of catharanthin (6) 13

Figure S13: BPC and MS profile of tabersonine (7).. 14

Figure S14: BPC and MS profile of yohimbine (8)... 14

Figure S15: BPC and MS profile of geissoschizine (9) 15

Figure S16: BPC and MS profile of quebrachidine (vincarine) (10)... 15

Figure S17: BPC and MS profile of pleiocarpamine (11) 16

Figure S18: BPC and MS profile of deacetylvindoline (12)... 16

Figure S19: BPC and MS profile of vindolinine (13)... 17

Figure S20: BPC and MS profile of tubotaiwine (14) 17

Figure S21: BPC and MS profile of alstonine (15).. 18

Figure S22: BPC and MS profile of coronaridine (16).. 18

Figure S23: BPC and MS profile of ajmalicine (17).. 19

Figure S24: BPC and MS profile of vindoline (18).. 19

Figure S25: BPC and MS profile of vindorosine (19).. 20

Figure S26: BPC and MS profile of vincristine (20).. 20

Figure S27: BPC and MS profile of vinformida (formyl leurosine) (21).. 21

Figure S28: BPC and MS profile of catharine (22).. 21

Figure S29: BPC and MS profile of vincaleukoblastine (23).. 22

Figure S30: BPC and MS profile of strychnine (24).. 22

Figure S31: BPC and MS profile of 2,3-dihydroxypropyl 9,12,15-octadecatrienoate (25)........................ 23

Figure S32: BPC and MS profile of linolenic acid (26).. 23

Figure S33: BPC and MS profile of oleanolic aldehyde (27).. 24

Figure S34: BPC and MS profile of ursolic acid (28).. 24

Figure S35: BPC and MS profile of 10(*S*)-hydroxypheophorbide a (29).. 25

Figure S36: BPC and MS profile of oleamide (30).. 25

Figure S37: BPC and MS profile of chlorin e_6 dimethylester (31).. 26

Figure S38: BPC and MS profile of pheophorbide a (32).. 26

Figure S39: BPC and MS profile of pyropheophorbide a (33).. 27

Figure S40: BPC and MS profile of methylpheophorbide a (34).. 27

Figure S41: Observed MS/MS profiles of the protonated molecular ions at *m/z* 353.268 (a)................... 28

and *m/z* 609.270 (b).. 28

Figure S42: Observed fragmentation pattern of 2,3-dihydroxypropyl 9,12,15-octadecatrienoate in (+)-ESI mode.. 29

Figure S43: Observed fragmentation pattern of (10*S*)-hydroxypheophorbide a in (+)-ESI mode............ 31

Figure S44: A graphical representation of Luminescence vs. concentration of *C. roseus* (Stem and Leaves).. 32

Table S1: Zone of inhibition of different extracts of *Catharanthus roseus*

S.N.	Extracts	*S. aureus*	*S. sonnei*	*K. pneumoniae*	*E. coli*
**1**	Positive Control	25	23	25	16
**2**	Negative Control	-	-	-	-
**3**	A01	12	14	11	-
**4**	A02	9	10	11	-
**5**	A03	9	9	9	-
**6**	A04	9	10	9	-
**7**	A05	12	13	10	-
**8**	A06	10	10	9	-

Table S2: MIC and MBC of different extracts against two bacterial strains

**Bacterial Strain**	**MIC/MBC (mg/mL)**									
	**A01**		**A02**		**A05**		**A06**		**Positive Control**	
	MIC	MBC	MIC	MBC	MIC	MBC	MIC	MBC	MIC	MBC
Staphylococcus aureus	12.5	25	12.5	25	12.5	25	12.5	25	0.004	0.01
Shigella sonnei	12.5	25	12.5	25	25	50	25	50	0.01	0.02

Table S3: Calculation of LC_{50} of methanolic (leaves) (A01) extract of *C. roseus*

Concentration (μg/mL)	Log C	Number of nauplii taken	Total number of surviving nauplii after 24 h	Total number of dead nauplii	Mortality %	Probit of Kill	LC_{50} (μg/mL)
**1000**	3.00	30	12	18	60	5.25	914.98
**800**	2.90	30	17	13	43.33	4.82	
**500**	2.70	30	17	13	43.33	4.82	
**250**	2.40	30	20	10	33.33	4.56	
**125**	2.10	30	20	10	33.33	4.56	
**100**	2.00	30	23	7	23.33	4.26	
**50**	1.70	30	22	8	26.68	4.39	

Table S4: Composition of artificial seawater

S.N.	Composition	Amount (g/L)
**1**	Na_2SO_4	4.00
**2**	NaCl	23.50
**3**	H_3BO_3	0.027
**4**	KCl	0.68
**5**	$MgCl_2.2H_2O$	10.68
**6**	$CaCl_2.2H_2O$	1.48
**7**	Na_2EDTA	0.0003
**8**	$NaHCO_3$	0.197

Table S5: Analysis of variance of DPPH radical scavenging activity

Source of Variation	SS	df	MS	F	P-value	F crit
Sample	1661.319	3	553.7731	17.23772	9.87E-08	2.798061
Columns	42358.77	5	8471.753	263.7068	1.13E-33	2.408514
Interaction	2118.564	15	141.2376	4.396413	4.32E-05	1.880175
Within	1542.031	48	32.12565			
Total	47680.68	71				

Table S6: post-hoc Tukey HSD test of DPPH radical scavenging activity

Comparison	Absolute difference	Critical value	Result
methanol to ethyl acetate	3.491393	5.02851	significantly not different
methanol to DCM	1.498775	5.02851	significantly not different
Methanol to Hexane	9.610869	5.02851	means are significantly different
Ethyl acetate to DCM	4.990167	5.02851	significantly not different
Ethyl Acetate to Hexane	13.10226	5.02851	means are significantly different
DCM to Hexane	8.112094	5.02851	means are significantly different

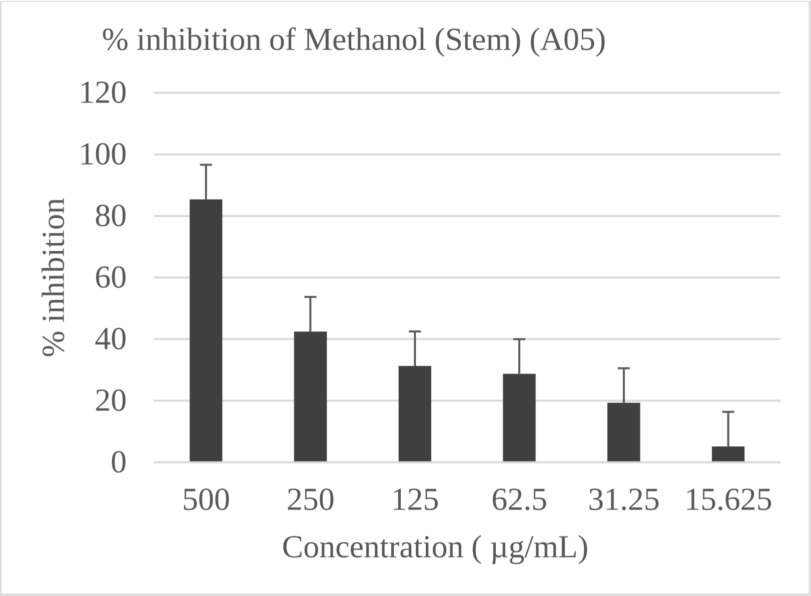

Figure S1: Plot of % inhibition of A05 (Methanolic extract of the stem) vs Concentration (µg/mL)

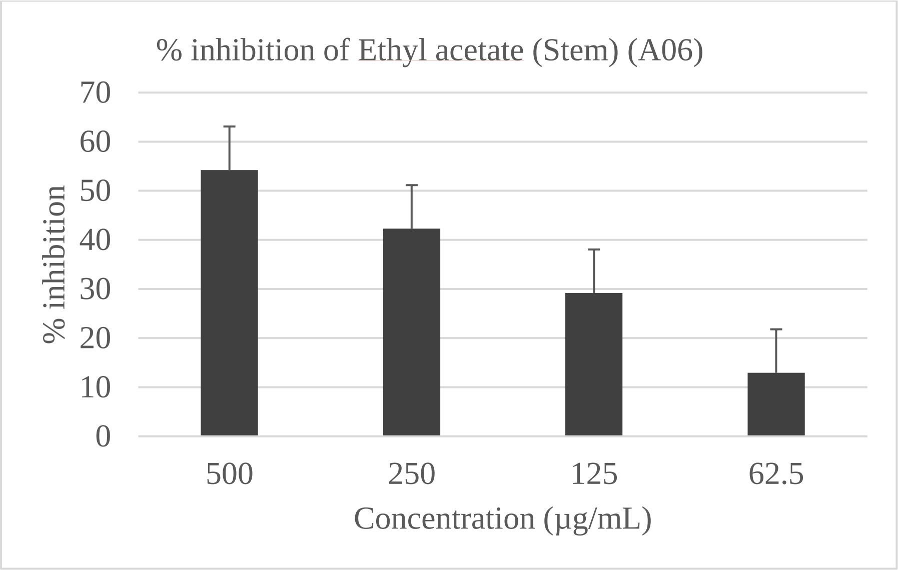

Figure S2: Plot of % inhibition of A06 (Ethyl acetate extract of the stem) vs Concentration (µg/mL)

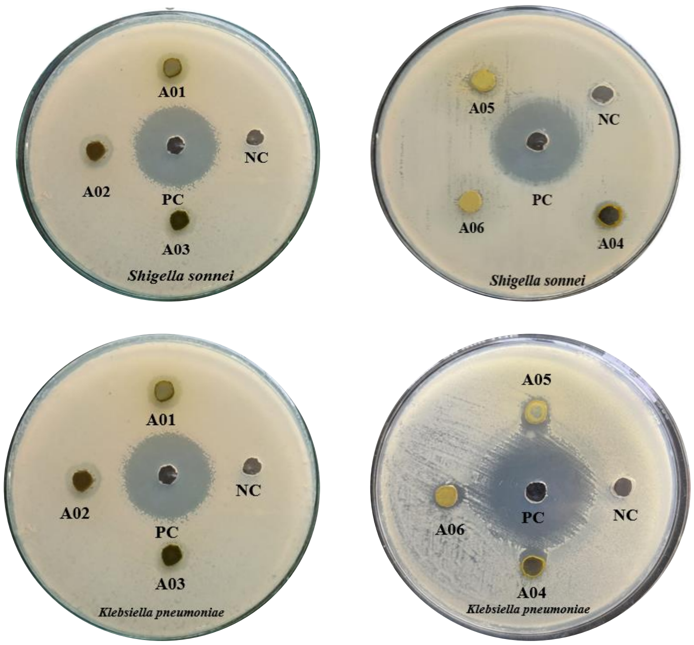

Figure S3: Antimicrobial activity of various extracts of *C. roseus* against *Shigella sonnei* and *Klebsiella pneumonia*

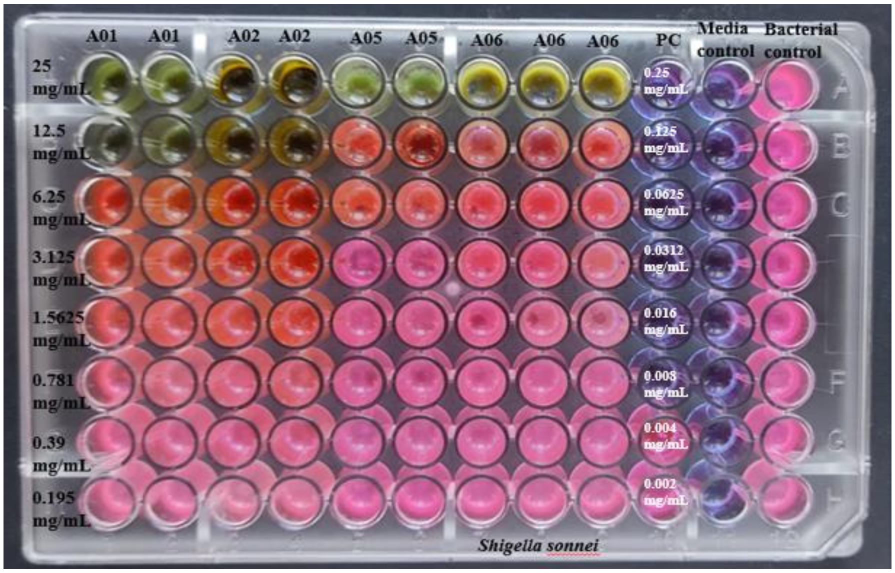

Figure S4: MIC of different extracts of *C. roseus* against *S. sonnei* (For PC, dilution was started from 0.25mg)

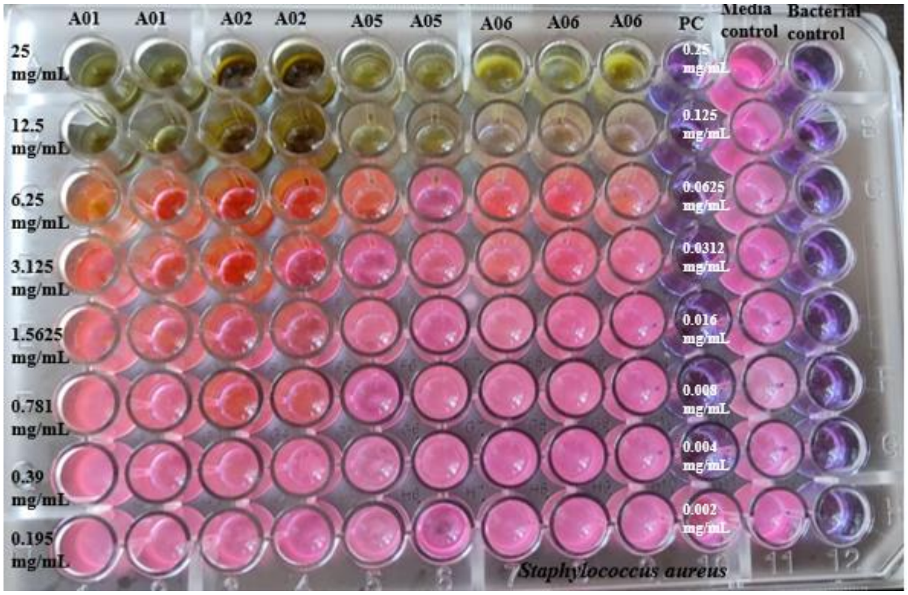

Figure S5: MIC of different extracts of *C. roseus* against *S. aureus* (For PC, dilution was started from 0.25mg)

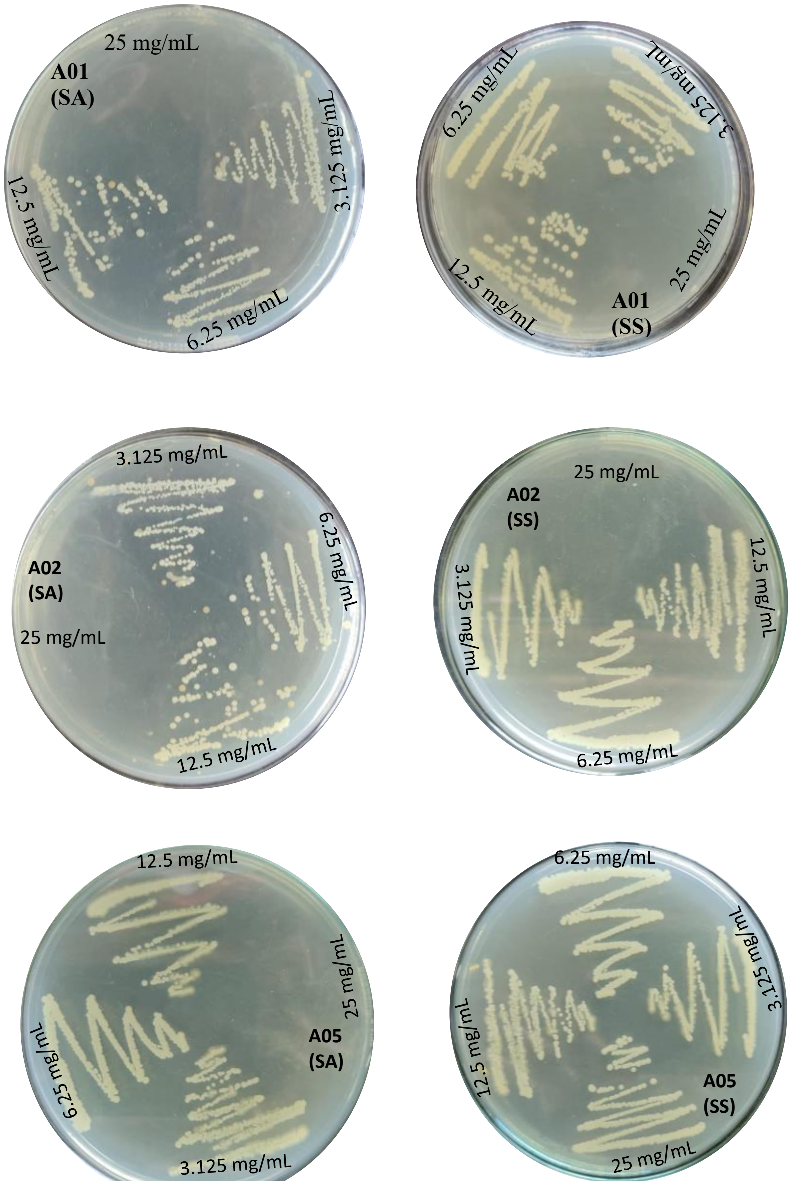
25 mg/mL
A01
(SA)
3.125 mg/mL
12.5 mg/mL
6.25 mg/mL
6.25 mg/mL
3.125 mg/mL
25 mg/mL
12.5 mg/mL
A01
(SS)
3.125 mg/mL
6.25 mg/mL
A02
(SA)
25 mg/mL
12.5 mg/mL
25 mg/mL
A02
(SS)
12.5 mg/mL
3.125 mg/mL
6.25 mg/mL
12.5 mg/mL
25 mg/mL
A05
(SA)
6.25 mg/mL
3.125 mg/mL
6.25 mg/mL
3.125 mg/mL
12.5 mg/mL
A05
(SS)
25 mg/mL

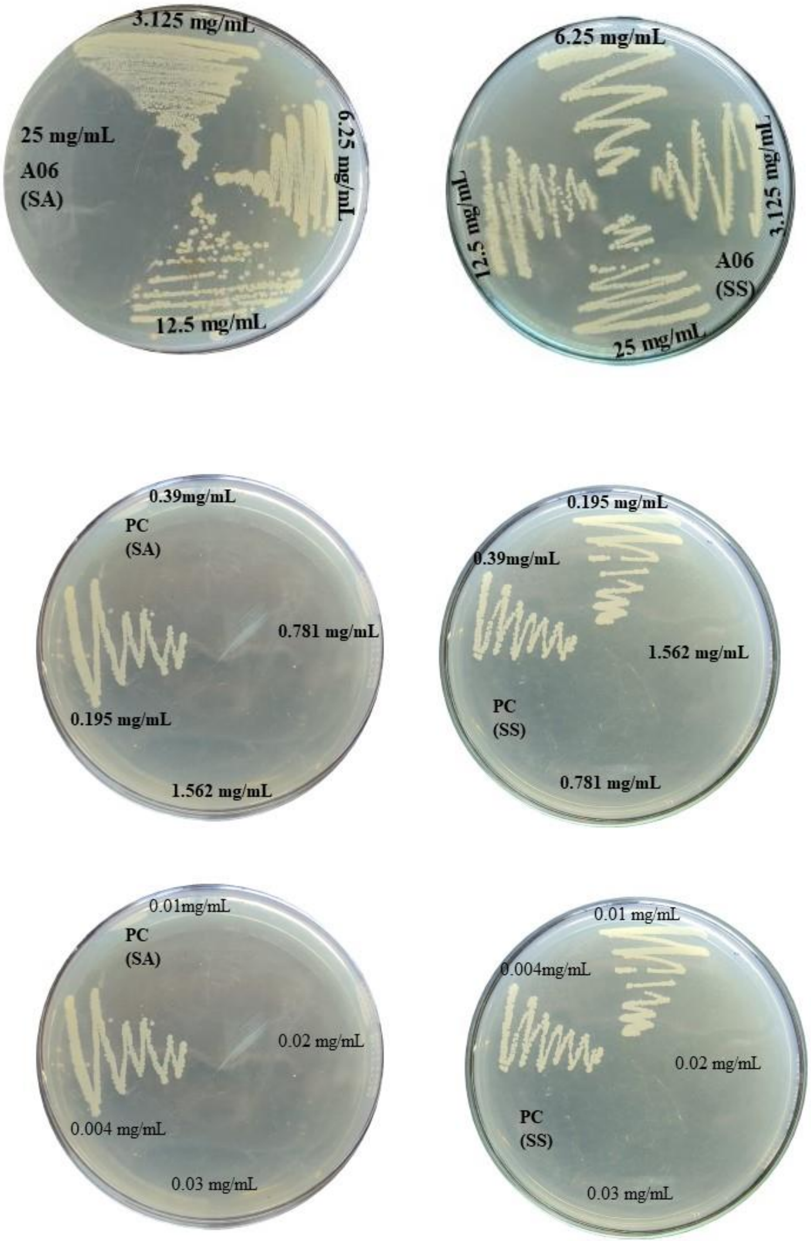

Figure S6: MBC of different extract of leaves and stem of *C. roseus* along with positive control against *S. aureus* in the right and *S. sonnei* in the left

Chromatogram of annotated compounds:

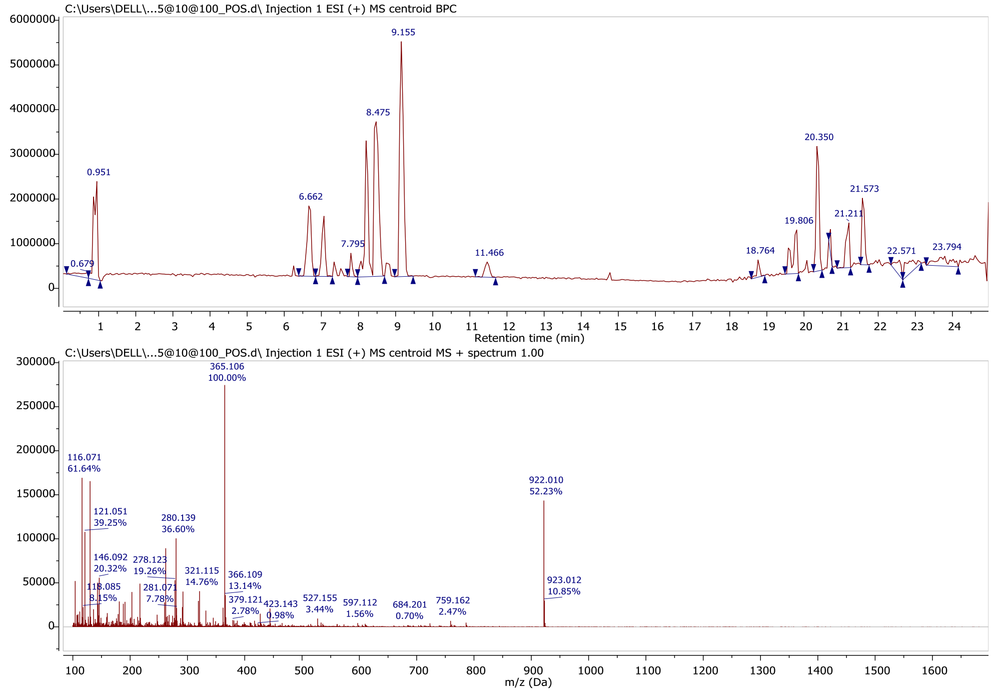

Figure S7: BPC and MS profile of L-proline (**1**)

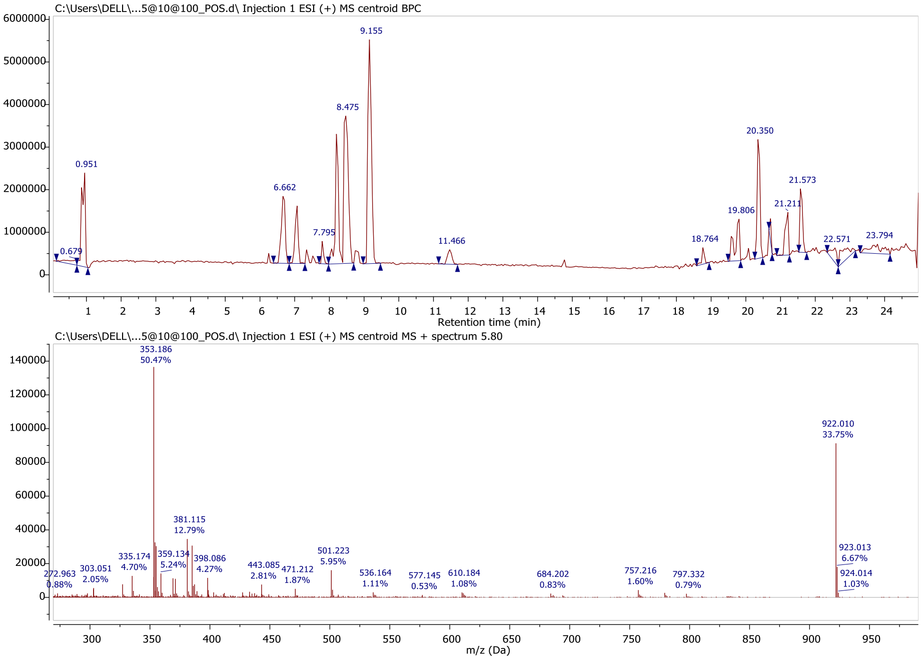

Figure S8: BPC and MS profile of preakuammicine (**2**)

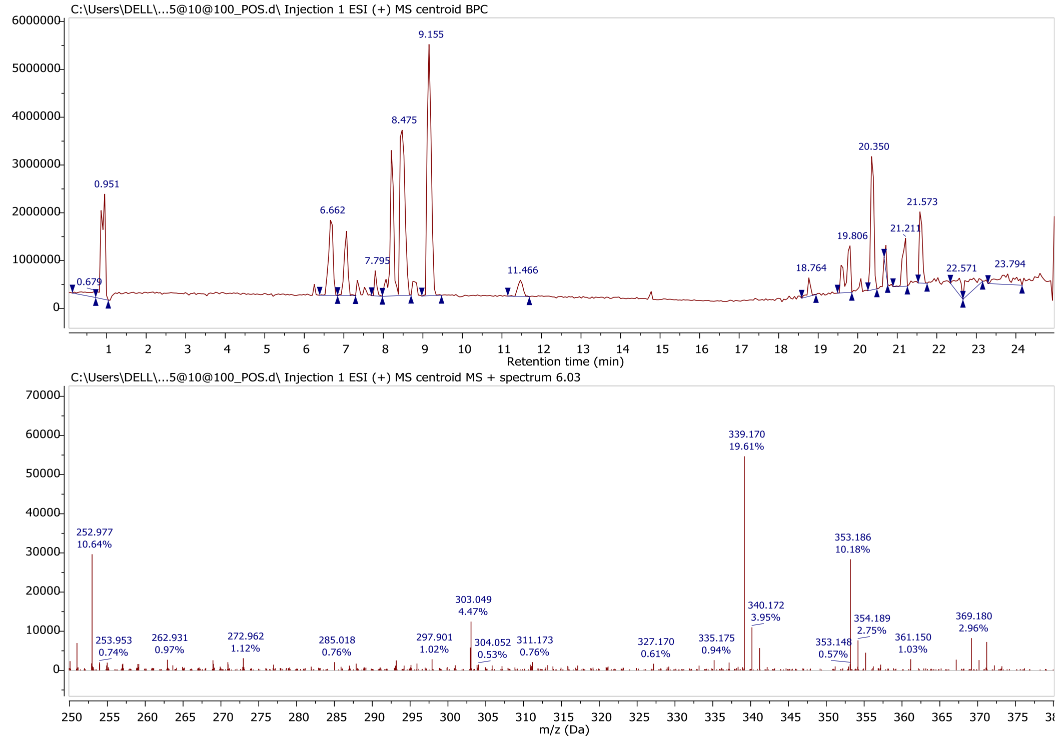

Figure S9: BPC and MS profile of quercetin (**3**)

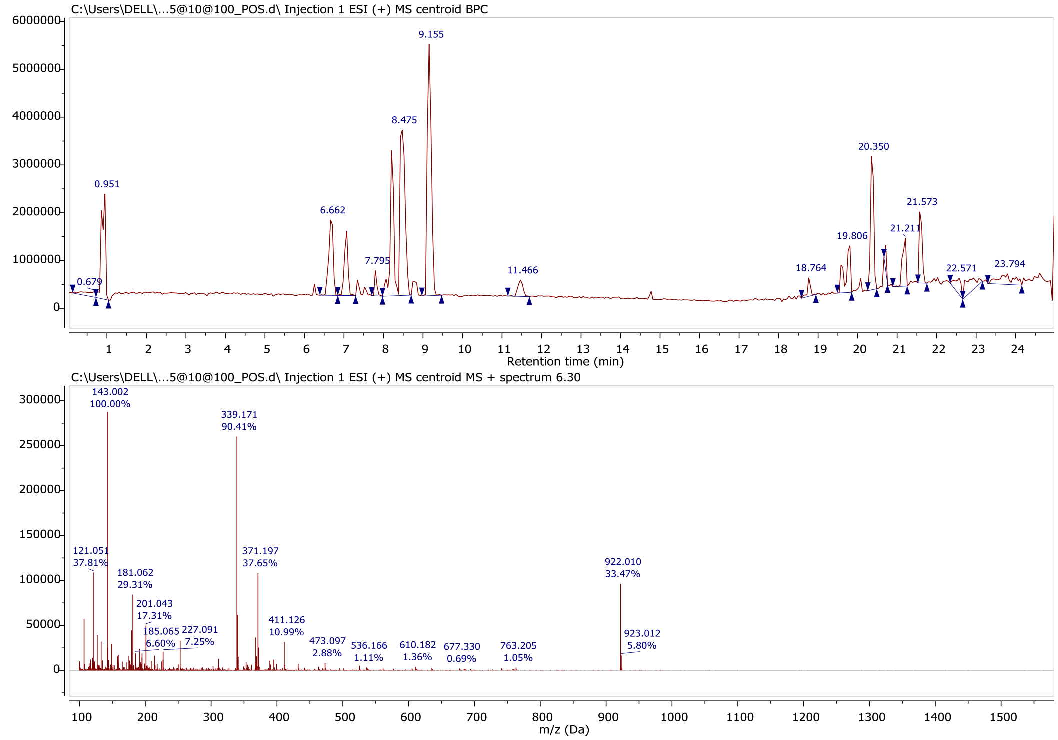

Figure S10: BPC and MS profile of perivine (**4**)

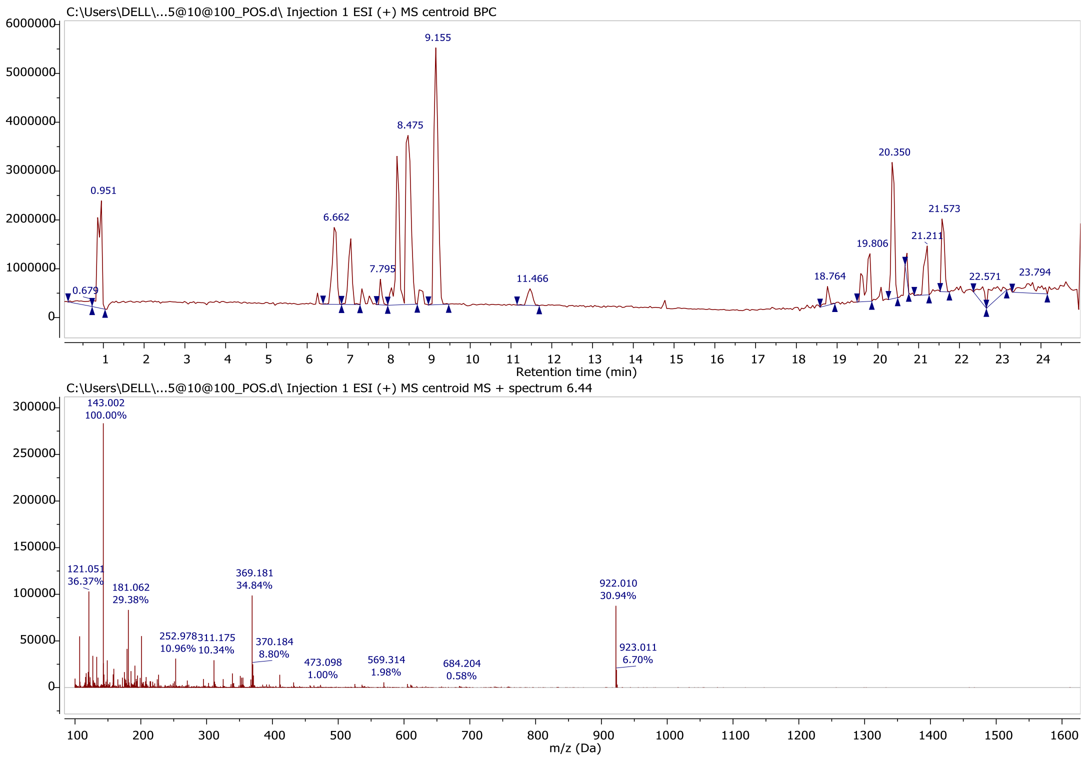

Figure S11: BPC and MS profile of mitraphylline (ajmalicine oxindole B) **(5)**

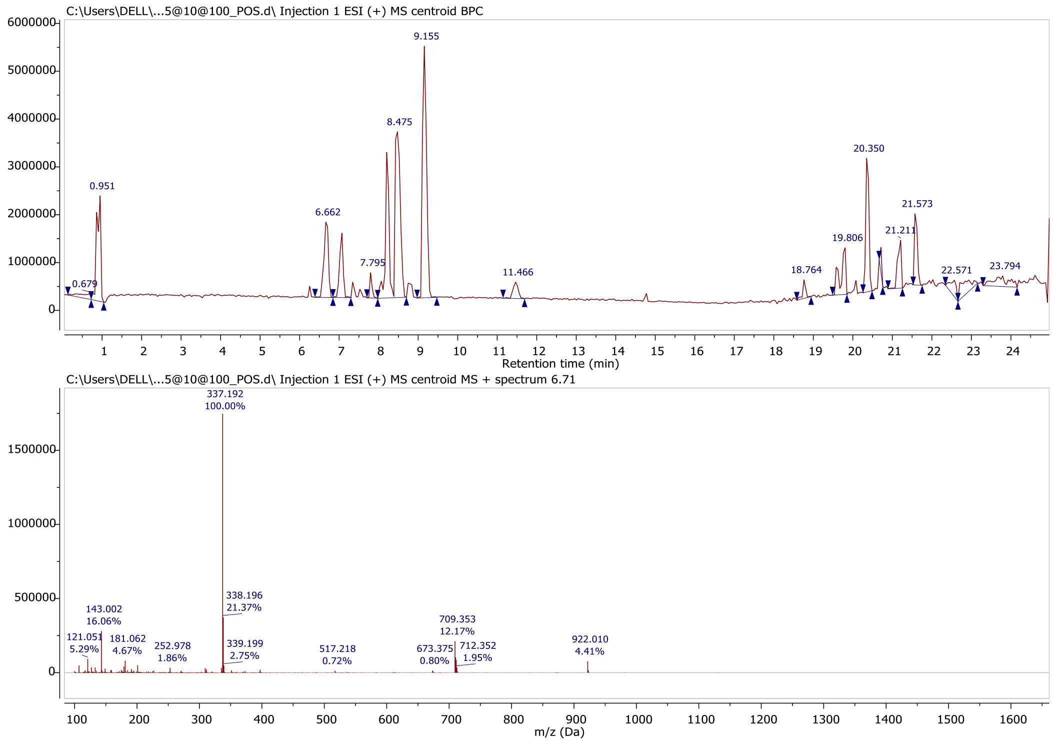

Figure S12: BPC and MS profile of catharanthin (6)

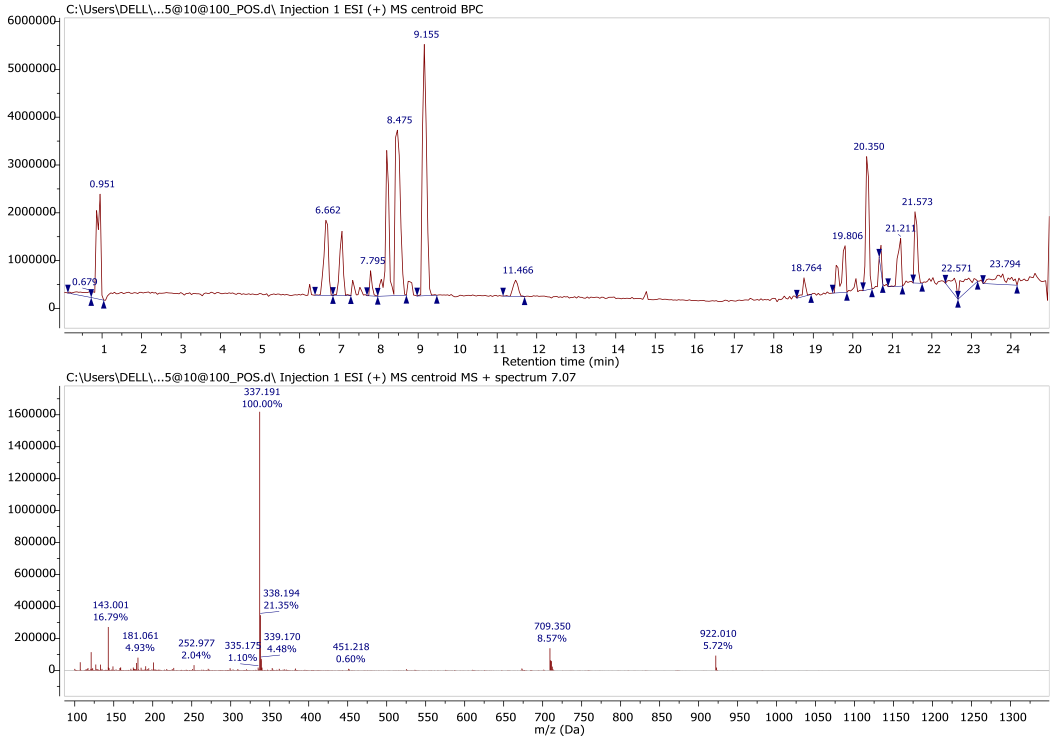

Figure S13: BPC and MS profile of tabersonine (**7**)

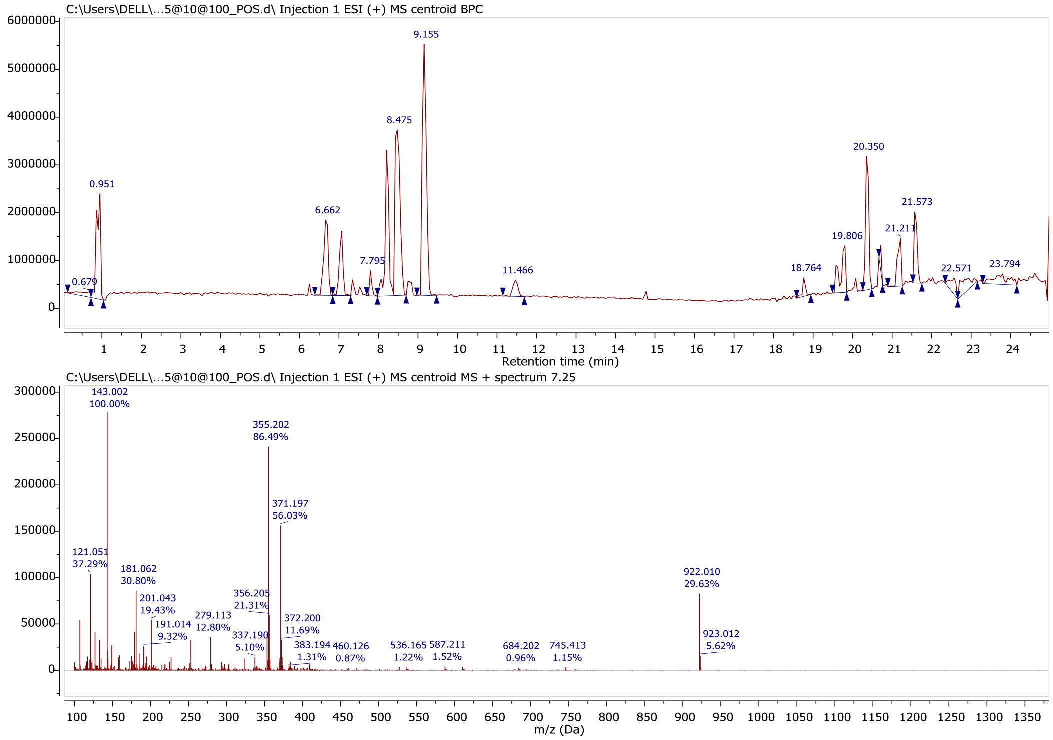

Figure S14: BPC and MS profile of yohimbine (**8**)

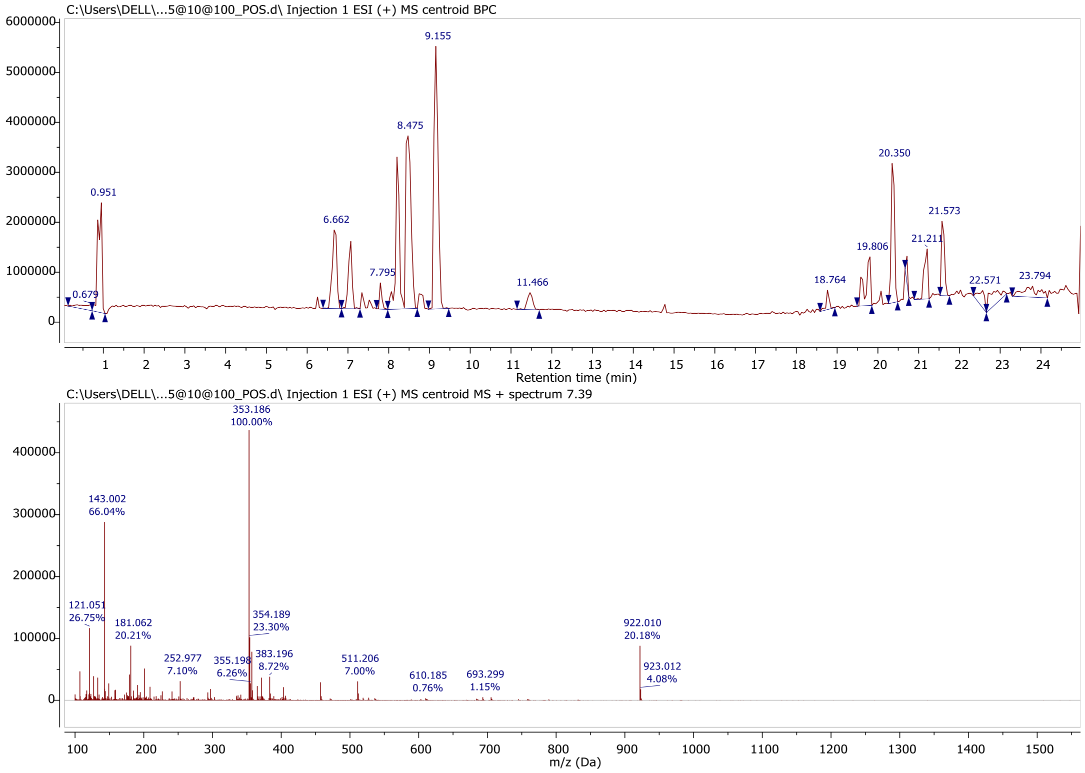

Figure S15: BPC and MS profile of geissoschizine (**9**)

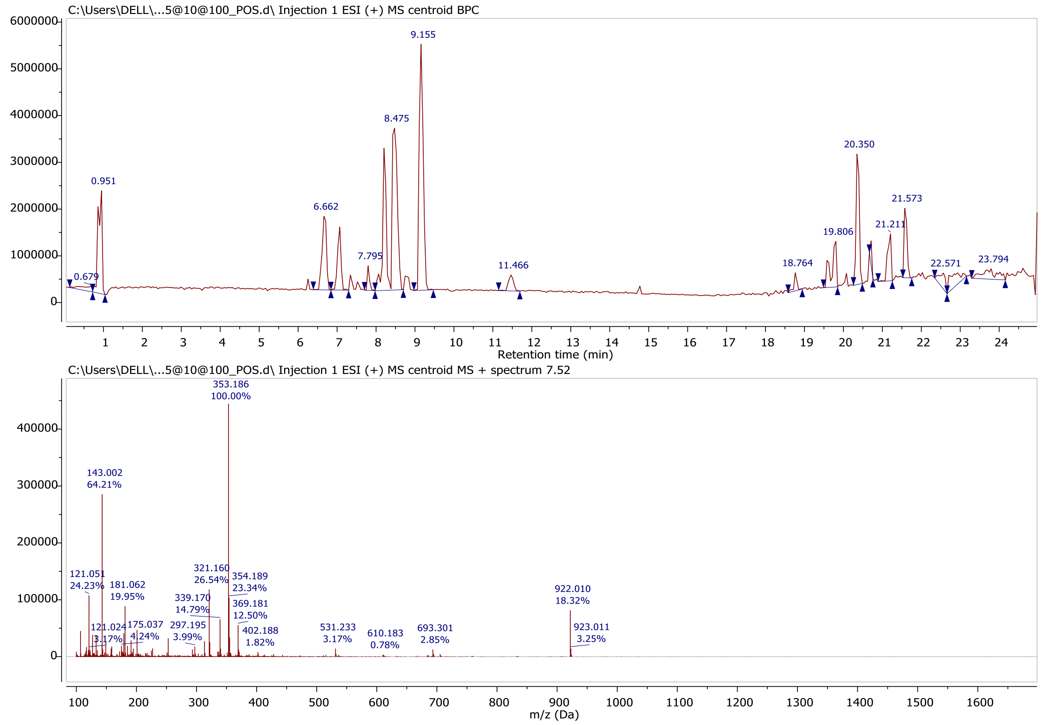

Figure S16: BPC and MS profile of quebrachidine (vincarine) (**10**)

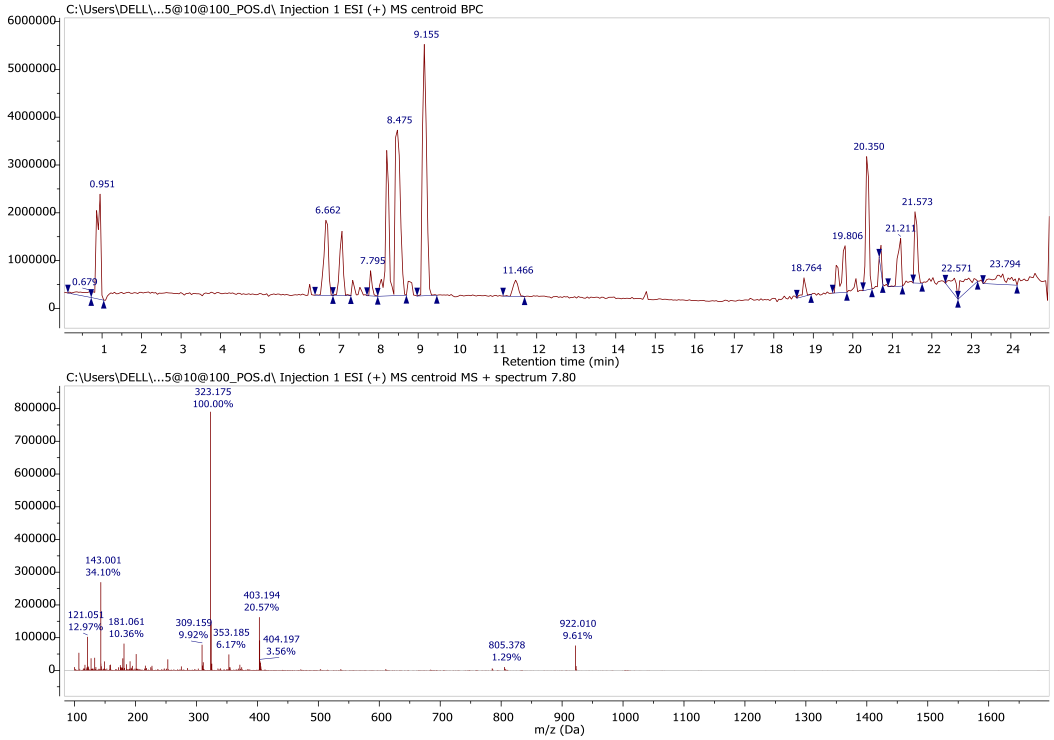

Figure S17: BPC and MS profile of pleiocarpamine (**11**)

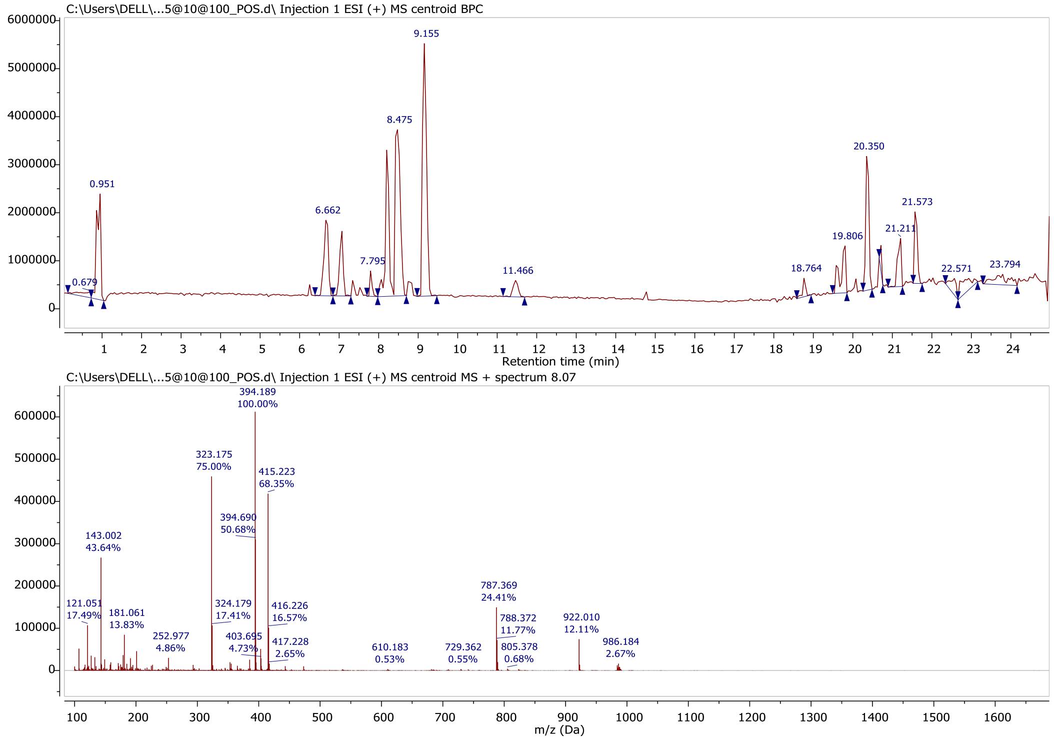

Figure S18: BPC and MS profile of deacetylvindoline (**12**)

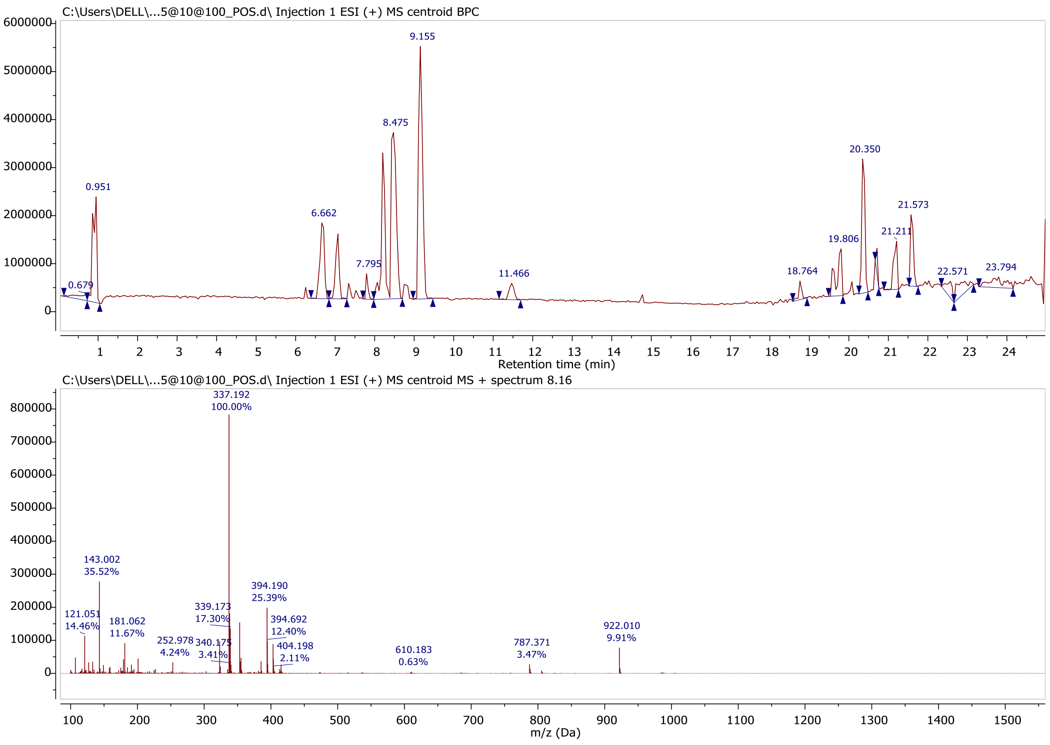

Figure S19: BPC and MS profile of vindolinine (**13**)

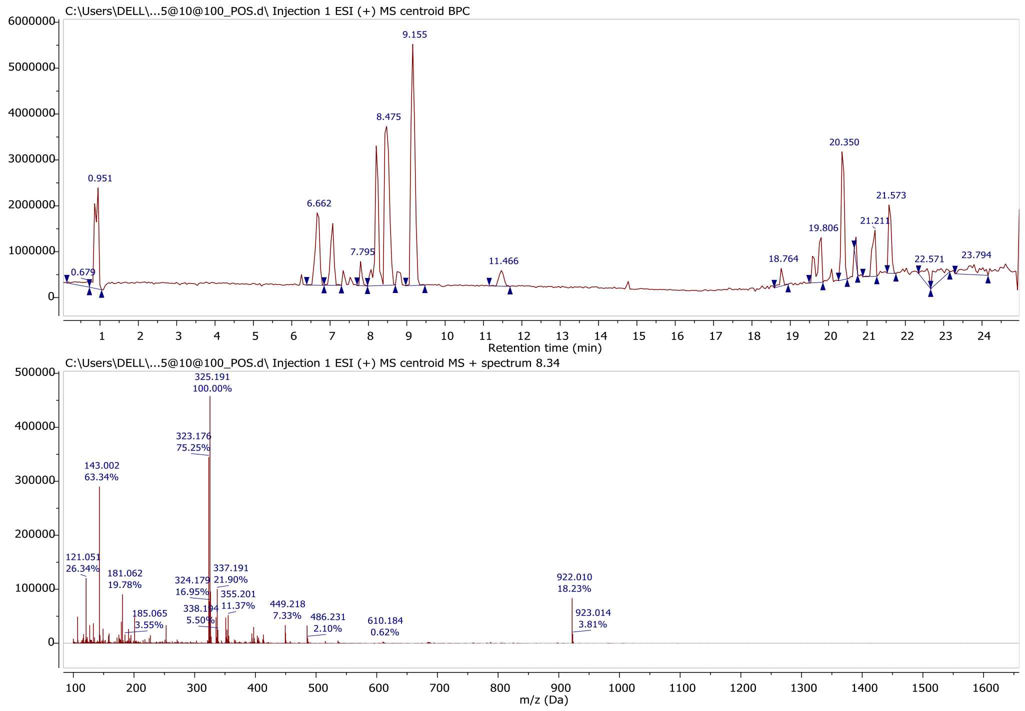

Figure S20: BPC and MS profile of tubotaiwine (**14**)

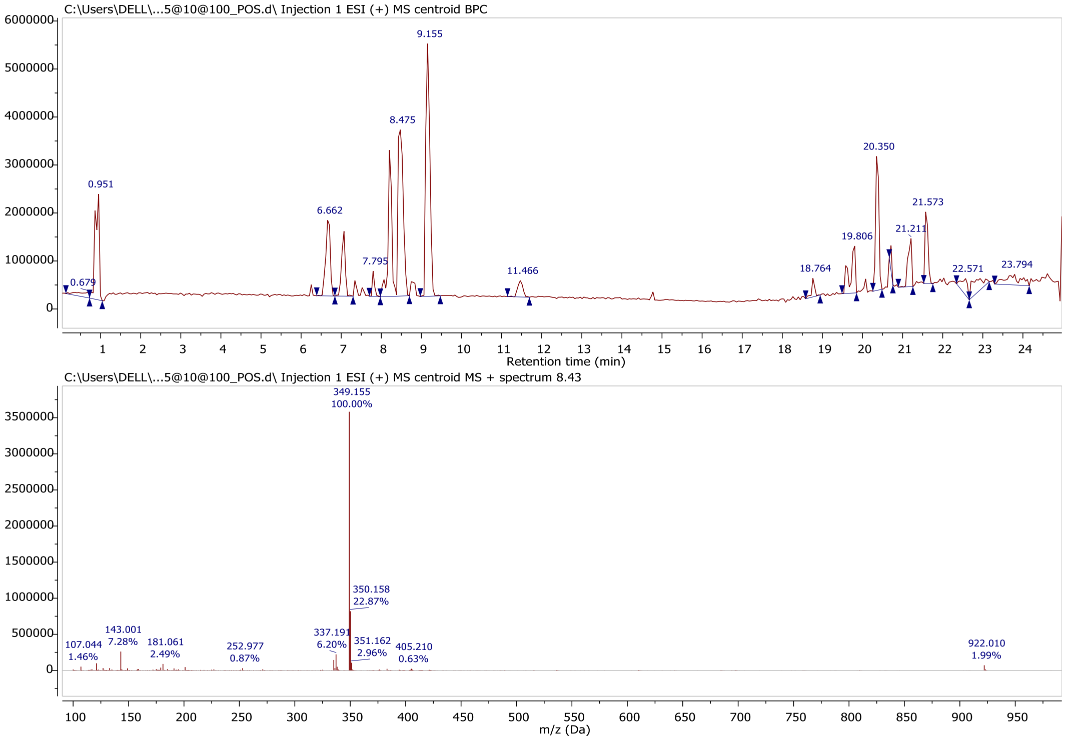

Figure S21: BPC and MS profile of alstonine (**15**)

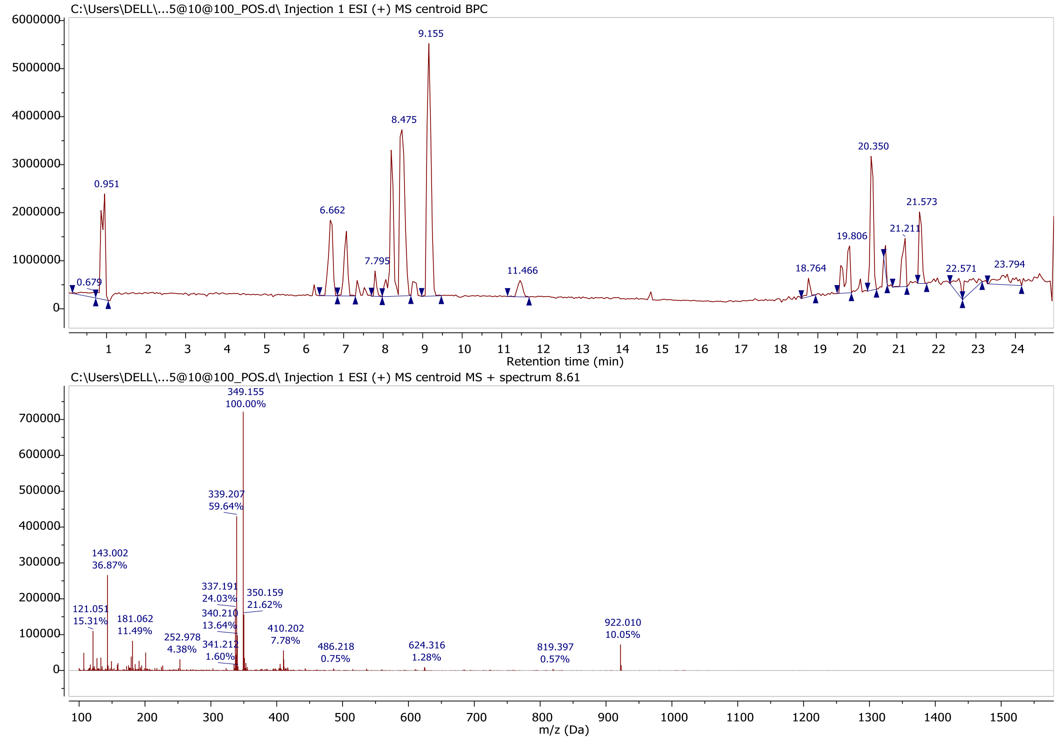

Figure S22: BPC and MS profile of coronaridine (**16**)

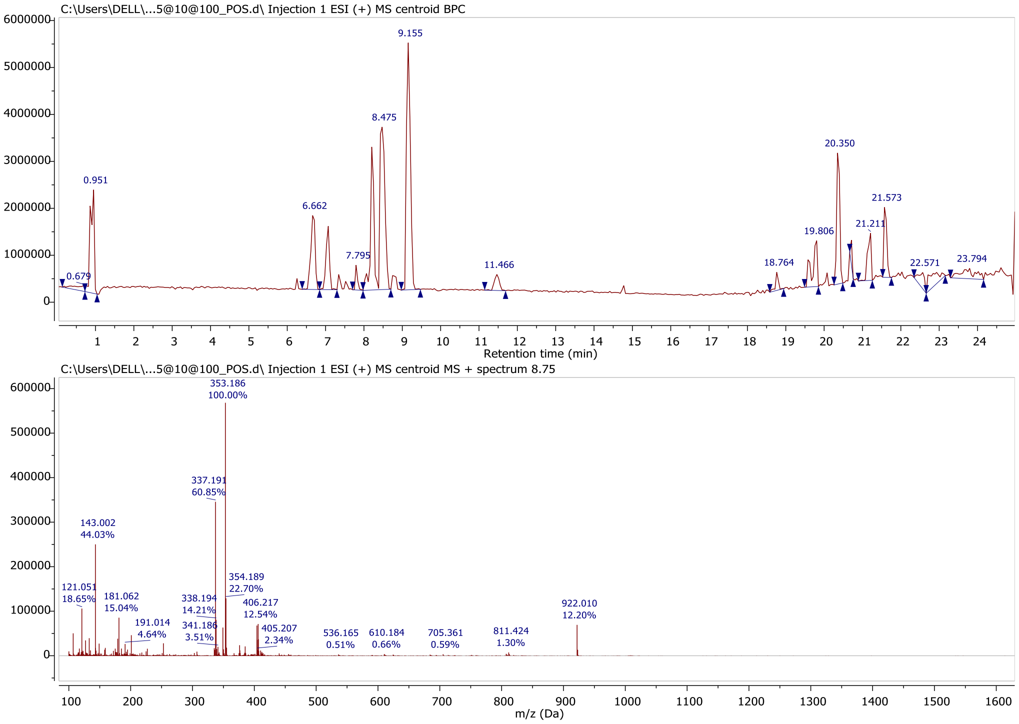

Figure S23: BPC and MS profile of ajmalicine (**17**)

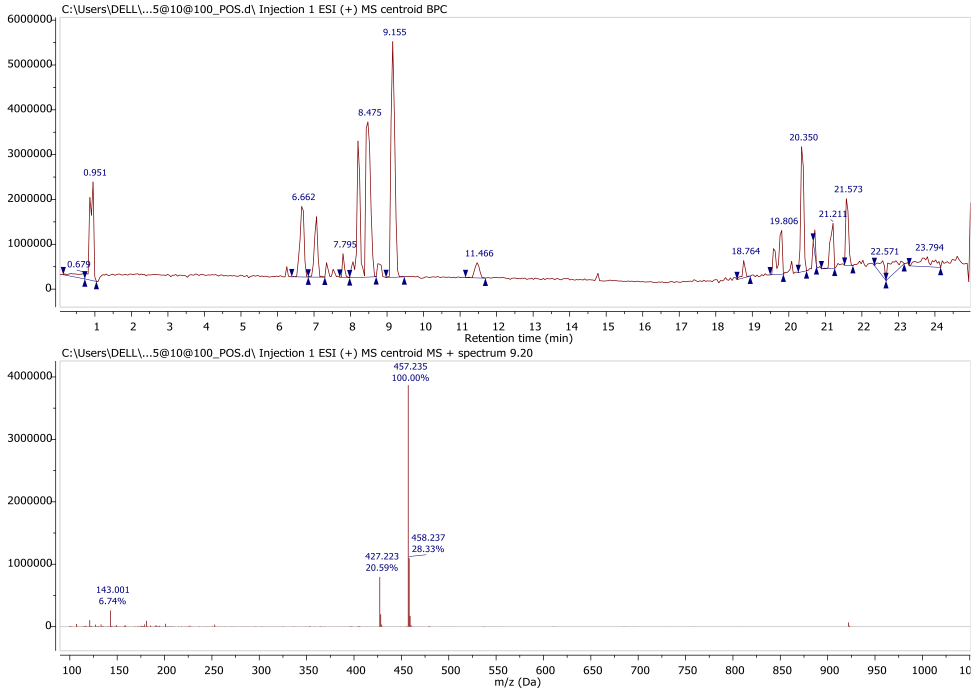

Figure S24: BPC and MS profile of vindoline (**18**)

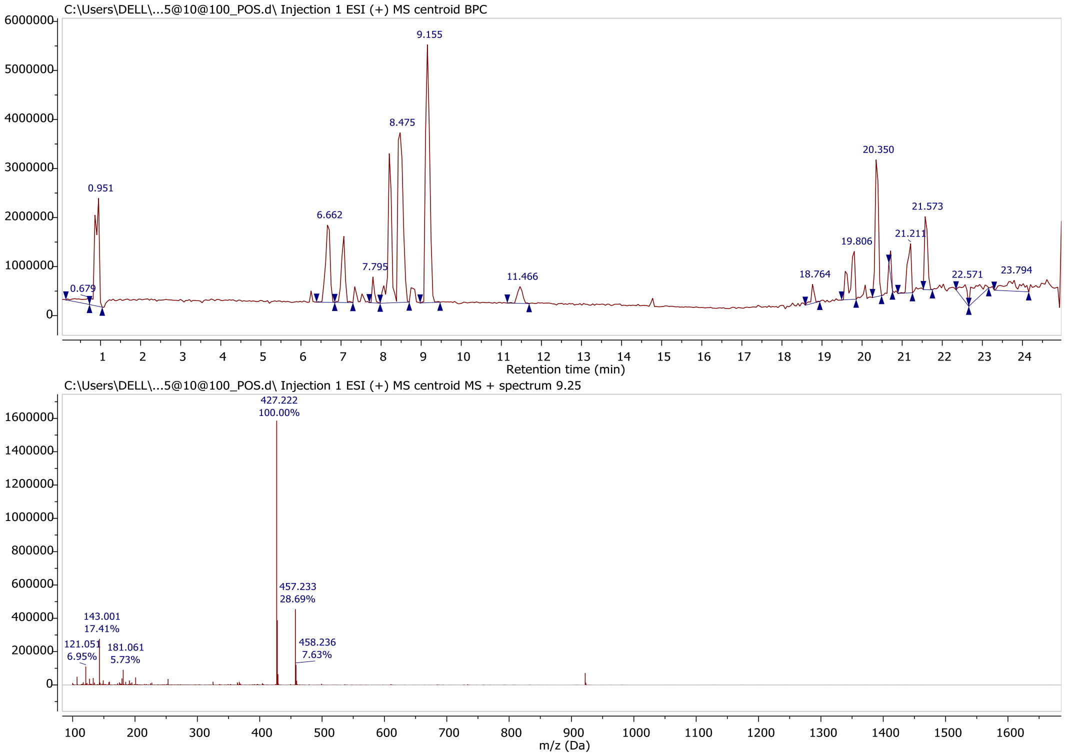

Figure S25: BPC and MS profile of vindorosine (**19**)

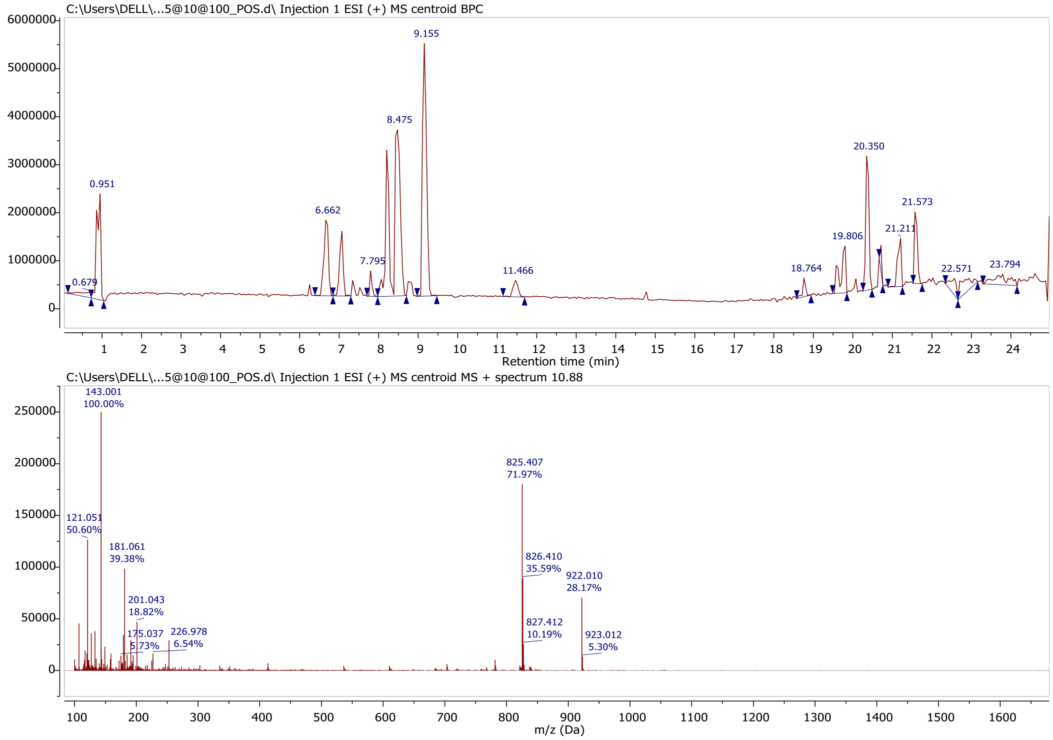

Figure S26: BPC and MS profile of vincristine (**20**)

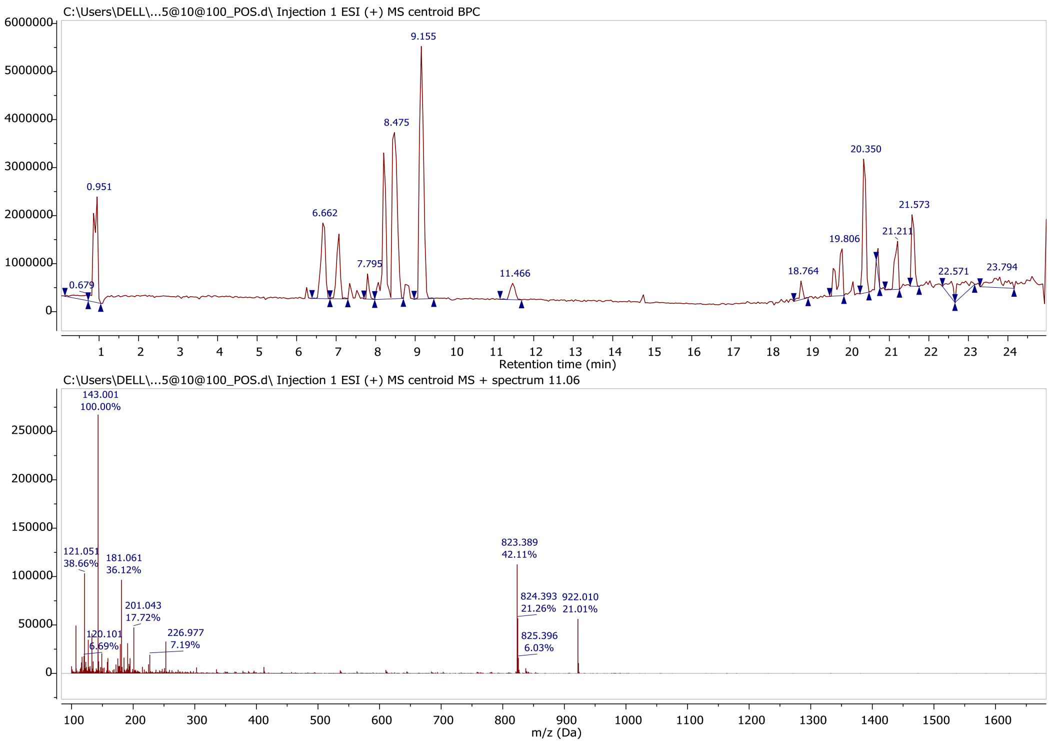

Figure S27: BPC and MS profile of vinformida (formyl leurosine) (**21**)

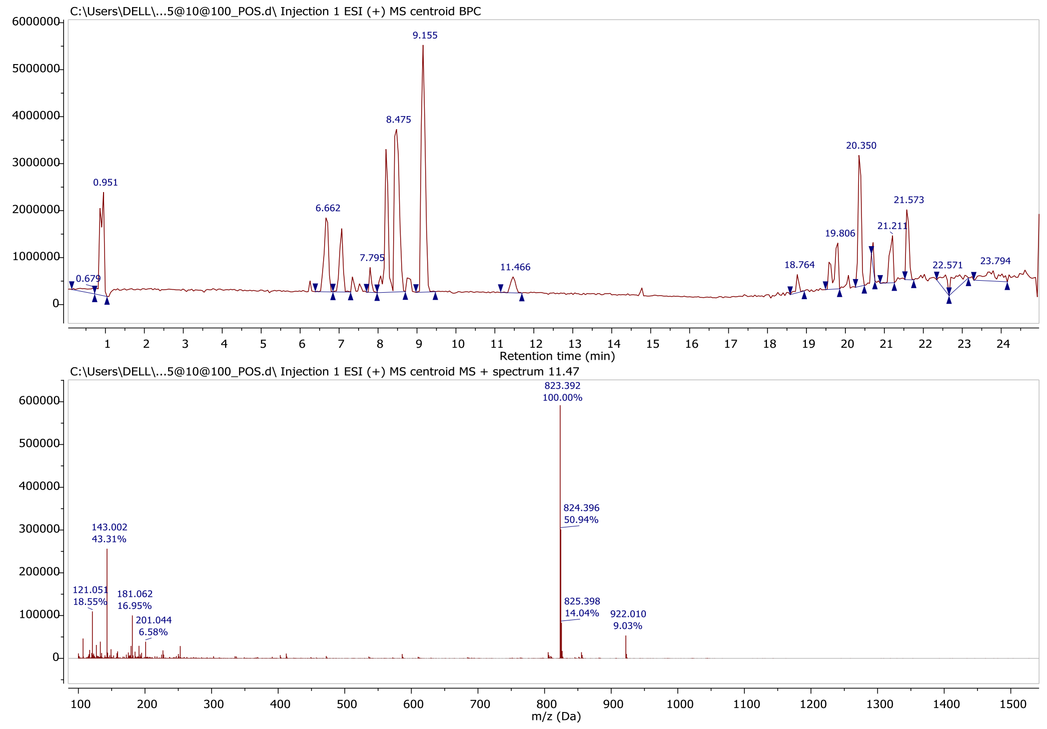

Figure S28: BPC and MS profile of catharine (**22**)

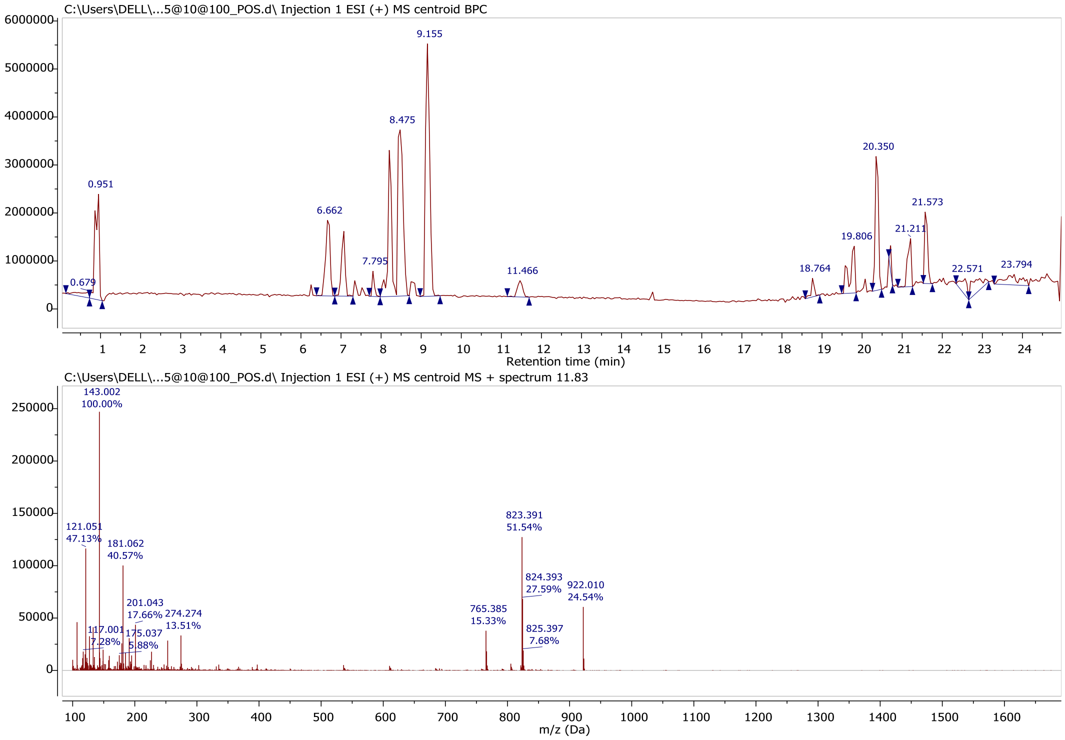

Figure S29: BPC and MS profile of vincaleukoblastine (**23**)

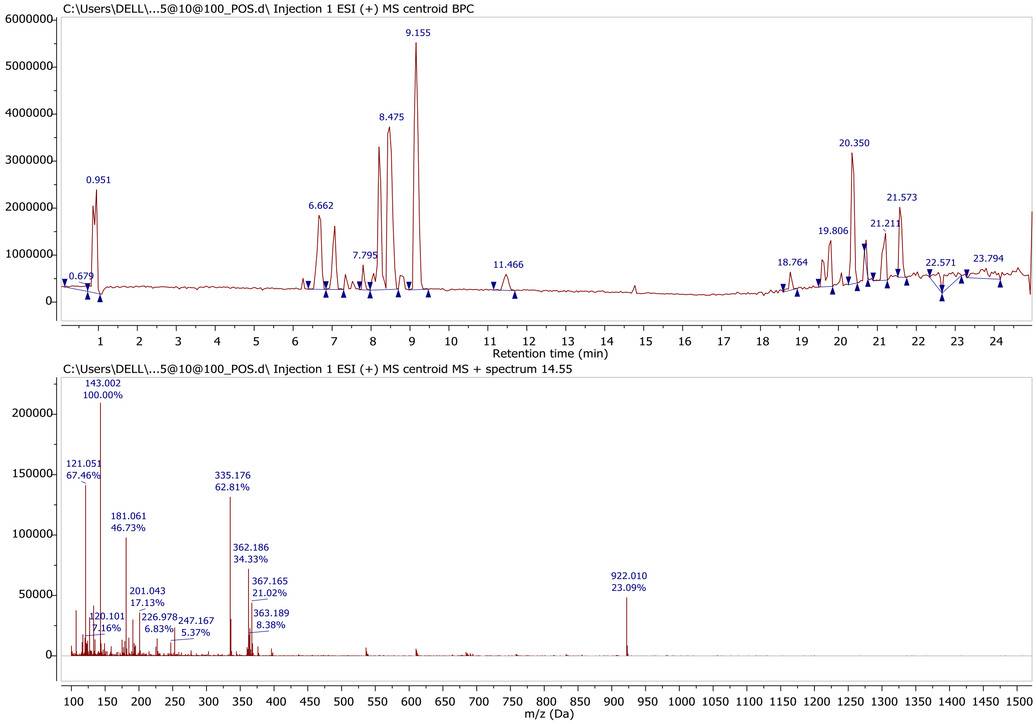

Figure S30: BPC and MS profile of strychnine (**24**)

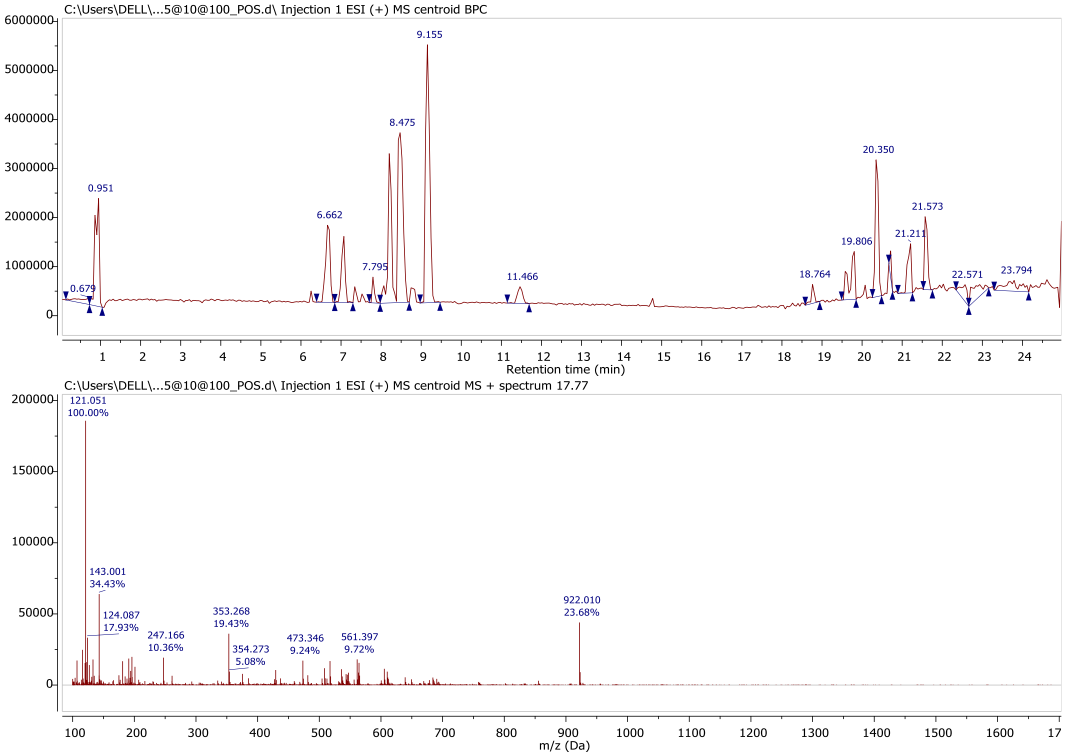

Figure S31: BPC and MS profile of 2,3-dihydroxypropyl 9,12,15-octadecatrienoate (**25**)

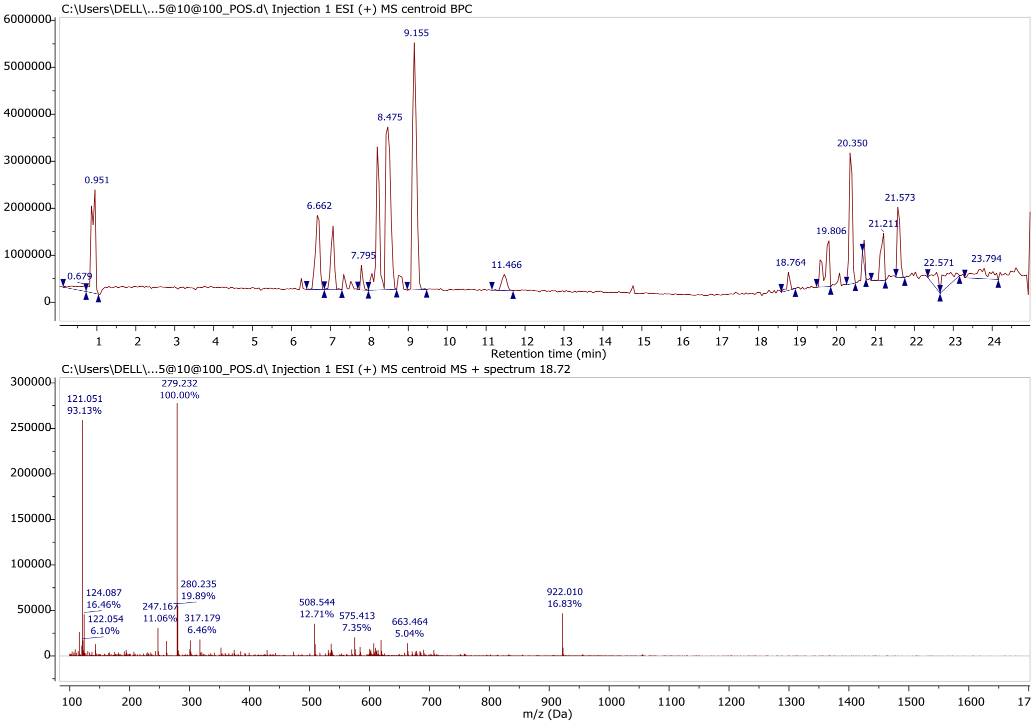

Figure S32: BPC and MS profile of linolenic acid (**26**)

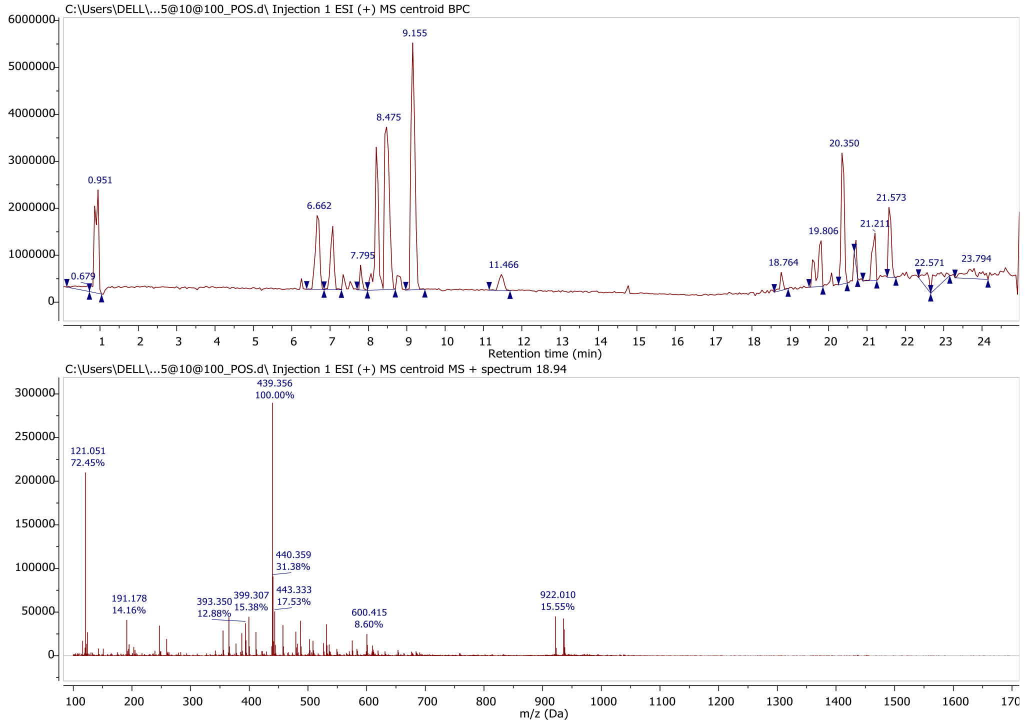

Figure S33: BPC and MS profile of oleanolic aldehyde (**27**)

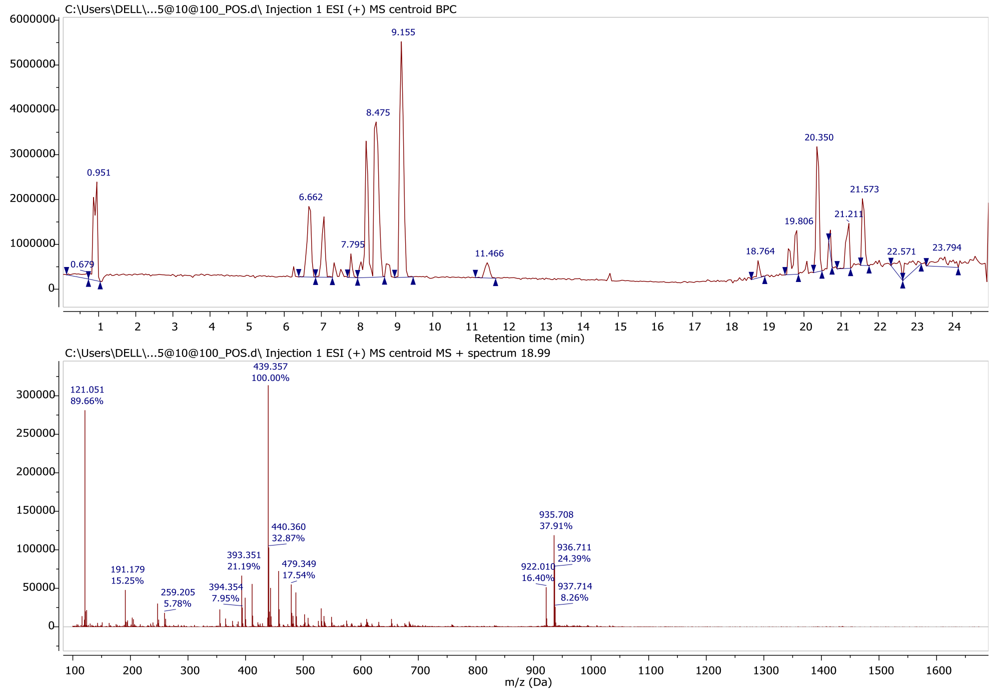

Figure S34: BPC and MS profile of ursolic acid (**28**)

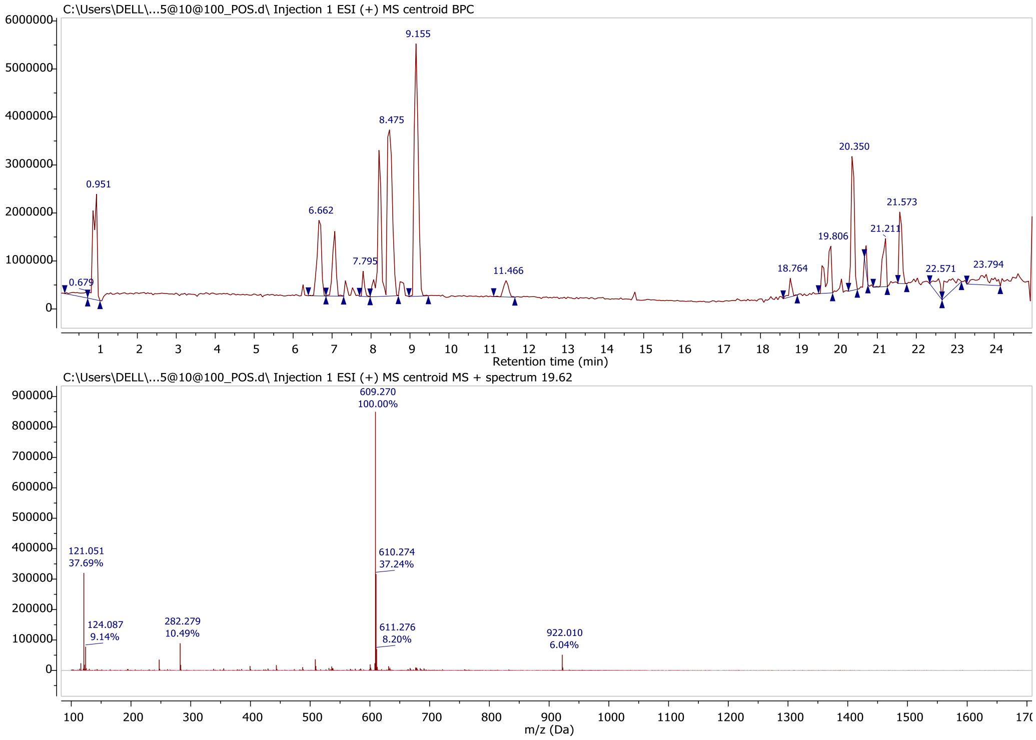

Figure S35: BPC and MS profile of 10(*S*)-hydroxypheophorbide a (**29**)

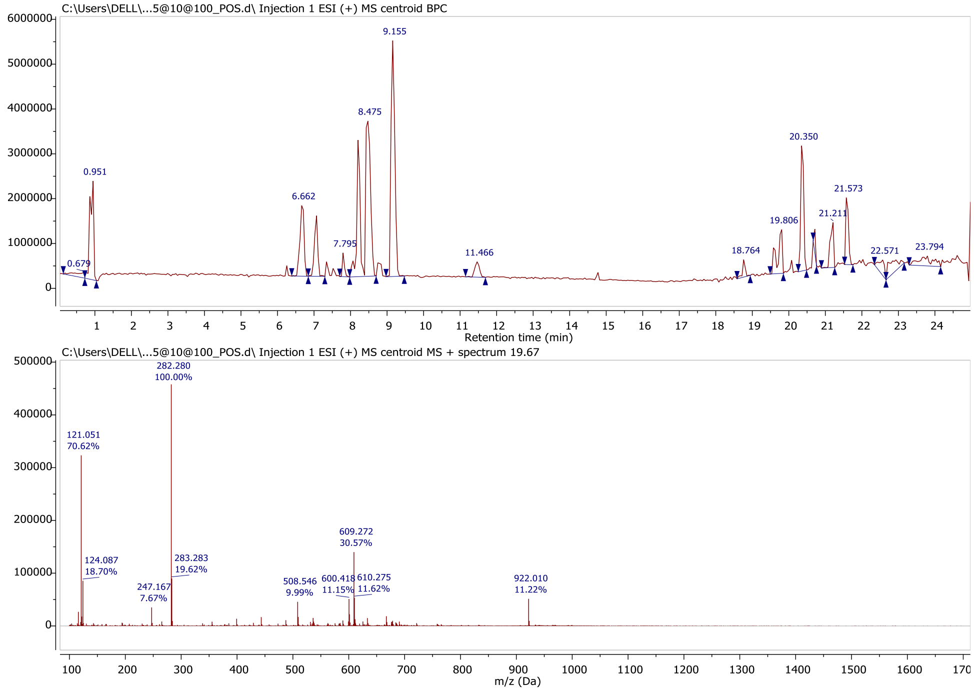

Figure S36: BPC and MS profile of oleamide (**30**)

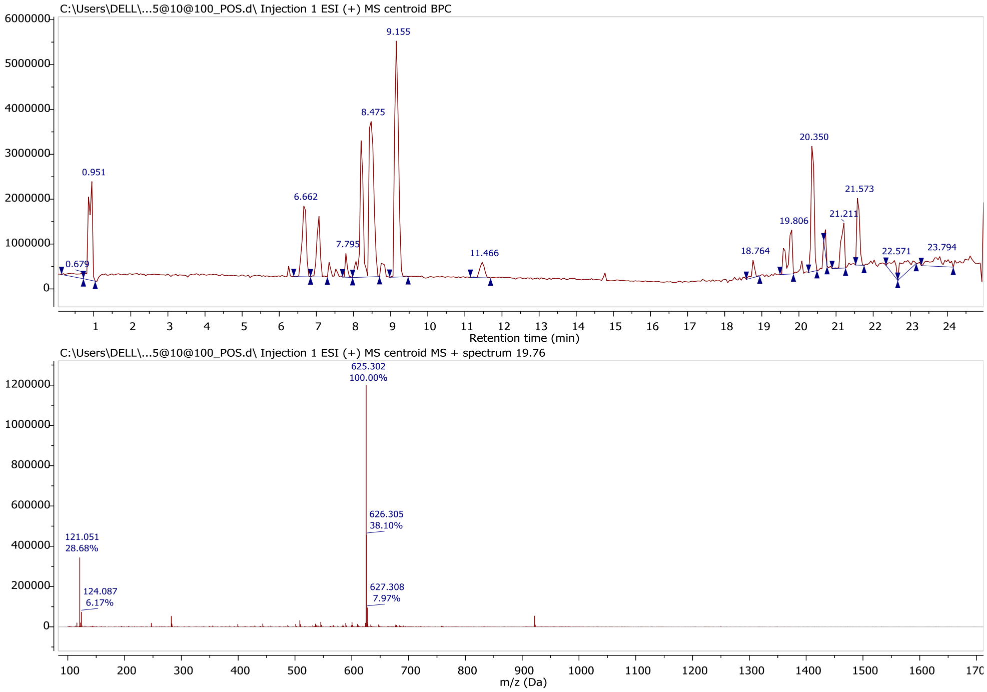

Figure S37: BPC and MS profile of chlorin e_6 dimethylester (**31**)

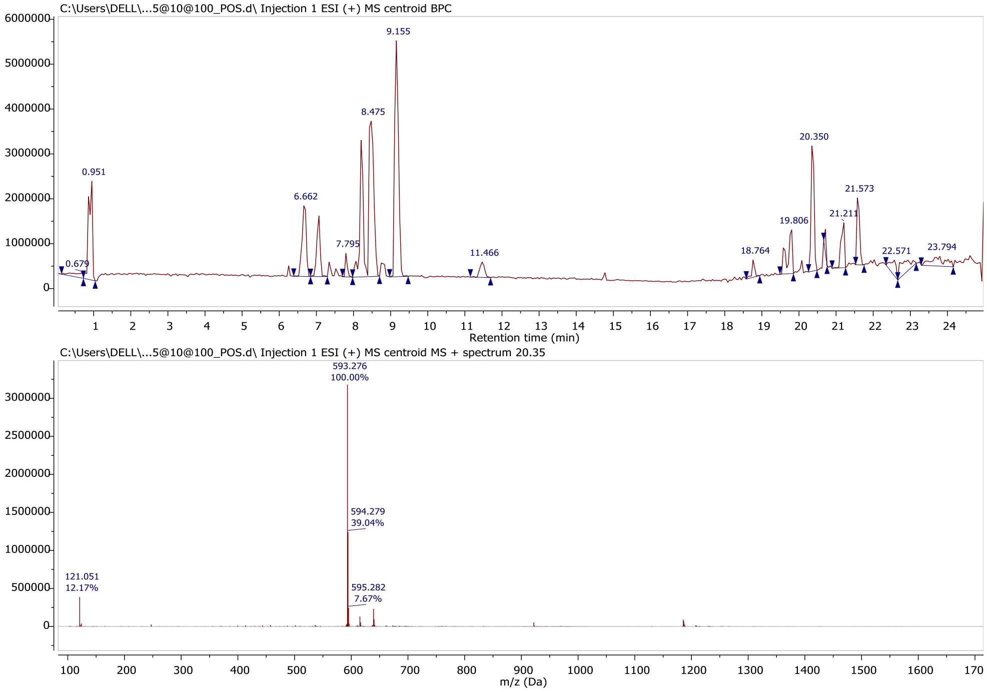

Figure S38: BPC and MS profile of pheophorbide a (**32**)

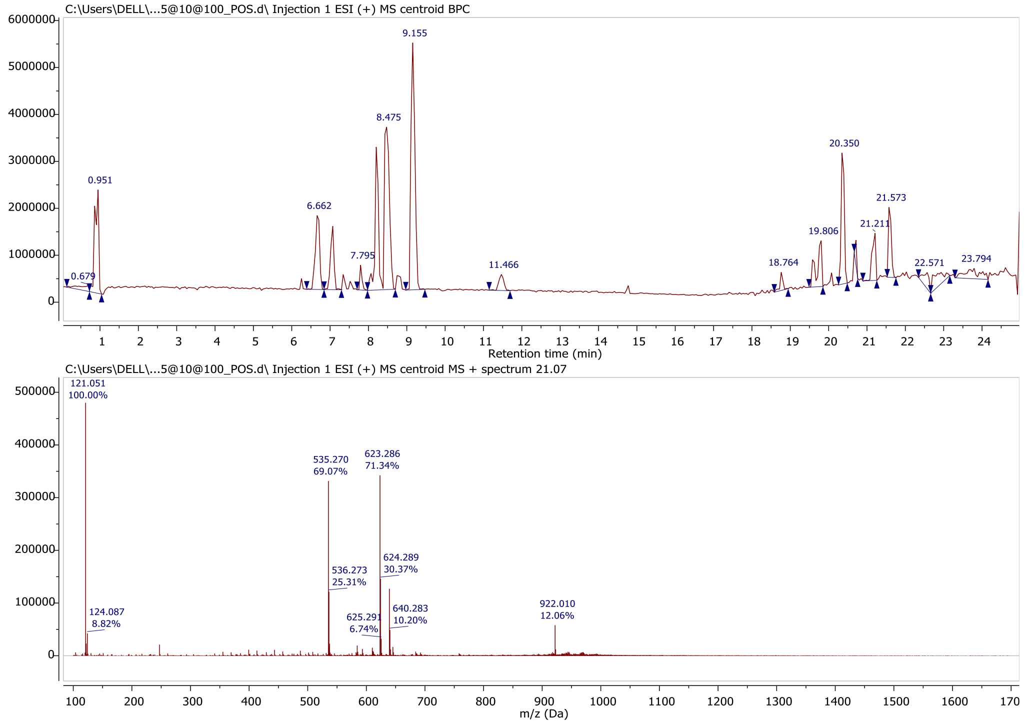

Figure S39: BPC and MS profile of pyropheophorbide a (**33**)

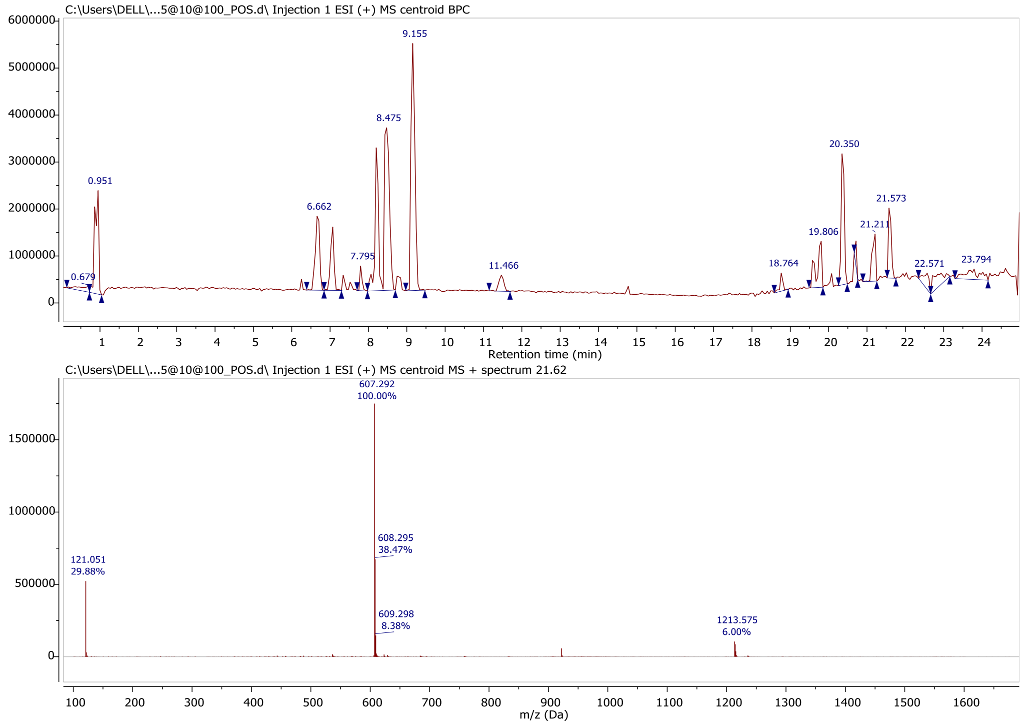

Figure S40: BPC and MS profile of methylpheophorbide a (**34)**

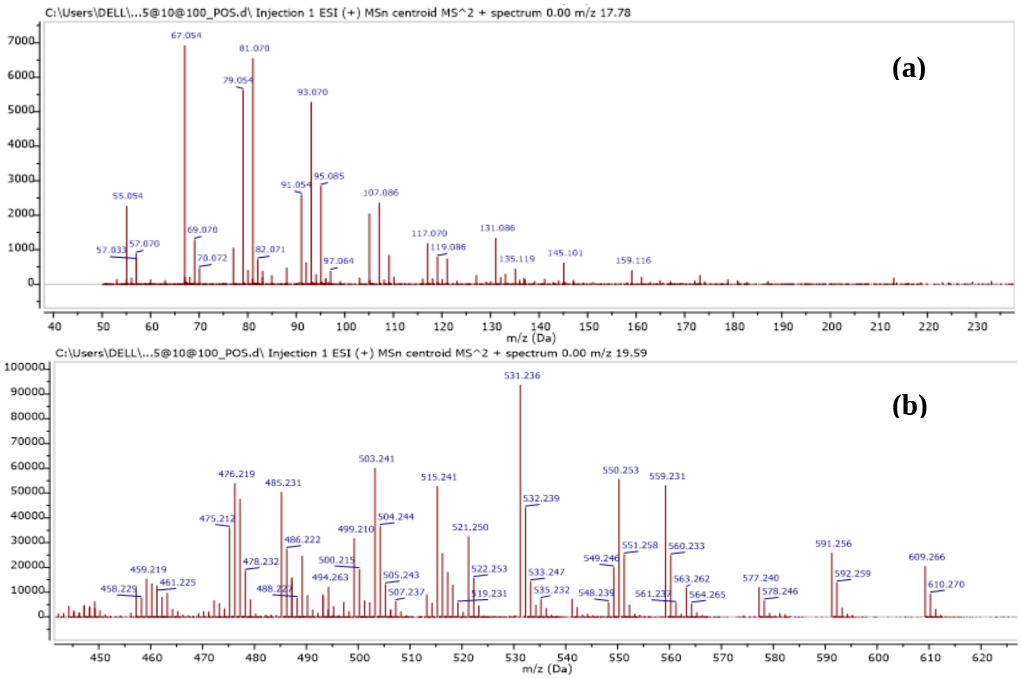

Figure S41: Observed MS/MS profiles of the protonated molecular ions at *m/z* 353.268 (**a**) and *m/z* 609.270 (**b**)

Among 34 metabolites, based on our information, two metabolites were identified for the first time in *Catharanthus roseus* species. The MS^2 spectrum of 2,3-Dihydroxypropyl 9,12,15-octadecatrienoate showed fragment ions at *m/z* 159.116 $[C_8H_{15}O_3]^+$ due to the departure of $[C_{13}H_{21}]^+$ and water simultaneously from a molecular ion peak detected at *m/z* 353.268 at a retention time of 17.77 min. Moreover, $[C_{13}H_{21}]^+$ further loses $[C_3H_7]^+$ and $[C_5H_9]^+$ forming a peak at *m/z* 67.05 $[C_5H_5]^+$. Similarly, $[C_8H_{15}O_3]^+$ ion loses a neutral methane molecule, thereby forming a peak at *m/z* 145.101 $[C_7H_{13}O_3]^+$, this further loses a methyl cation and forms a peak at *m/z* 131.07 as shown in **Figure S42**.

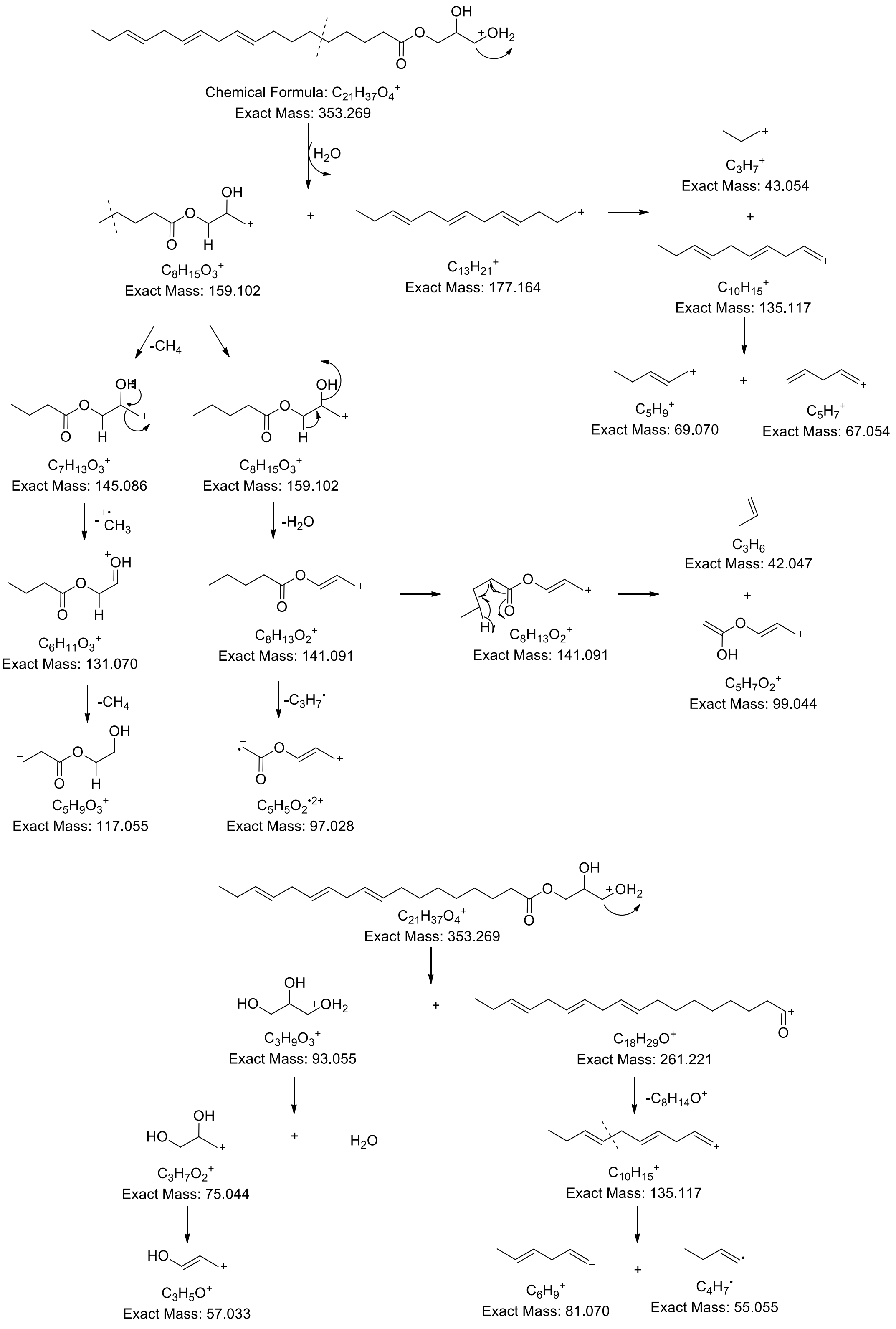

Figure S42: Observed fragmentation pattern of 2,3-dihydroxypropyl 9,12,15-octadecatrienoate in (+)-ESI mode

A molecular ion peak $[M+H]^+$ detected at *m/z* 609.270 $[M+H]^+$ at a retention time of 19.58 min was identified as (10S)-Hydroxypheophorbide a. Its MS^2 profile displayed a distinct peak at *m/z* 591.256 $[M+H-H_2O]^+$ resulted due to the loss of water, 550.253 $[C_{33}H_{34}N_4O_4]^{\bullet+}$ resulted due to the loss of neutral molecule carbon monoxide and methyl cation simultaneously from the molecular ion peak after the departure of water. Furthermore, $[C_{33}H_{34}N_4O_4]^{\bullet+}$ further lose a water molecule to give a distinct peak at *m/z* 531.236 attributed to $[C_{33}H_{31}N_4O_3]^{2\bullet+}$ ion. Similarly, $[C_{33}H_{31}N_4O_3]^{2\bullet+}$ loses a neutral CO forming a peak at *m/z* 503.241 as shown in **Figure S43**. The MS^2 spectrums of both these compounds are displayed in **Figure S41**.

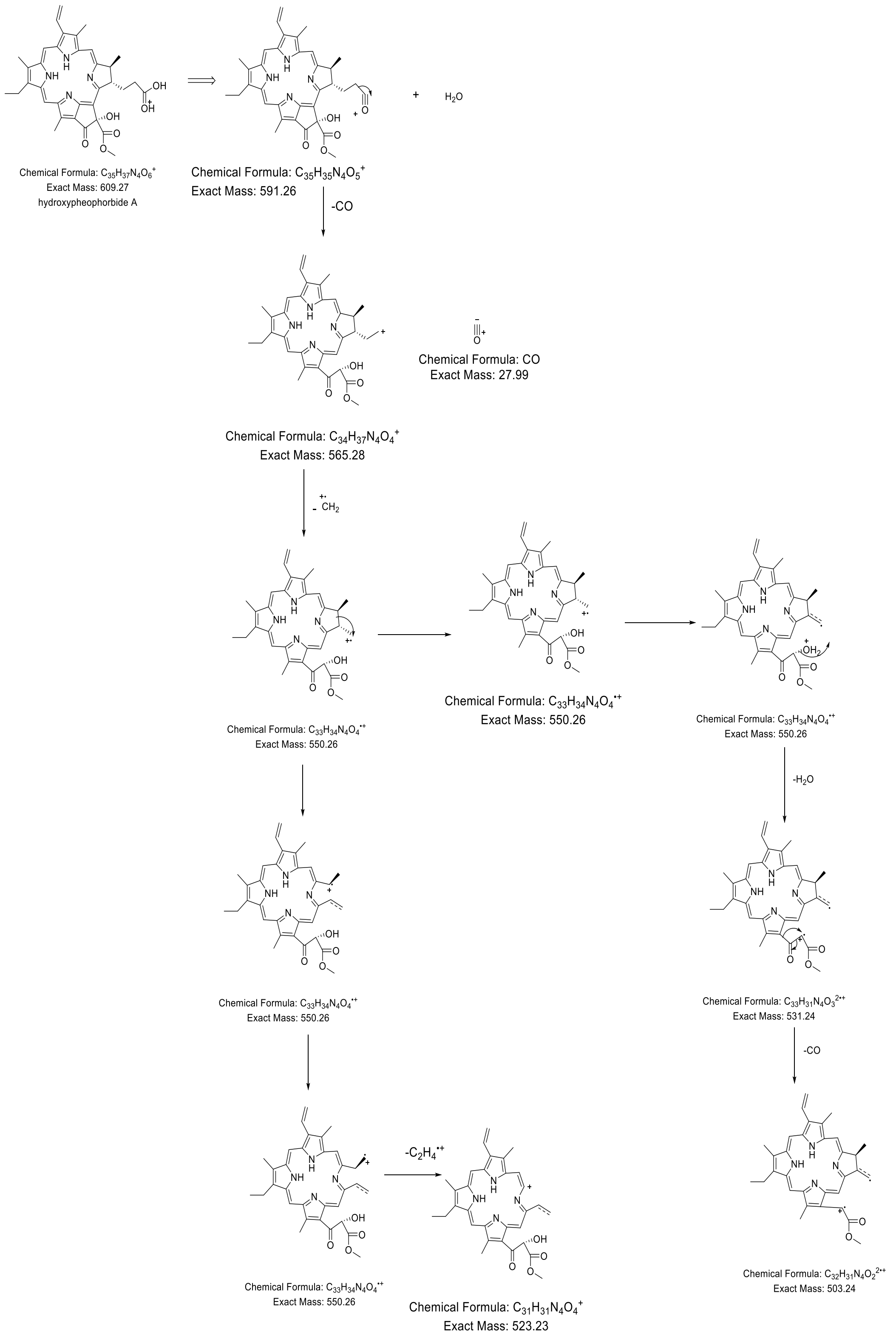

Figure S43: Observed fragmentation pattern of (10*S*)-hydroxypheophorbide a in (+)-ESI mode.

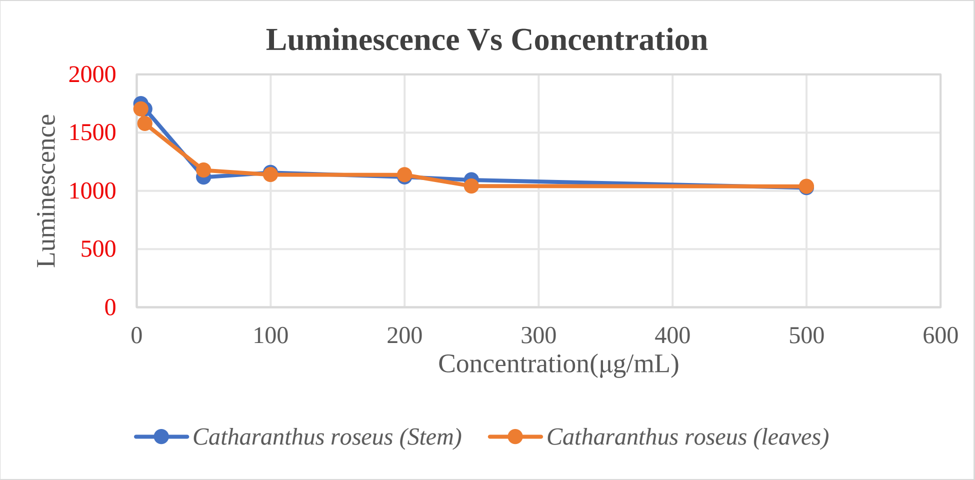

Figure S44: A graphical representation of Luminescence vs. concentration of *C. roseus* (Stem and Leaves)

Methodology

Plant Collection and Extract Preparation

For this purpose, 50 g of grounded plant (leaves) were soaked in 250 mL of respective solvents in four conical flasks for around 3 successive days with periodic shaking. Then, the solution was filtered. Furthermore, the solvent was added in the same amount after 48 h and 24 h, respectively, following the same procedure. The combined filtrates were kept in a water bath at around 40° C. Upon complete evaporation of the solvent, all the respective extracts were collected at different sample vials. For the stem, the process was repeated using methanol and ethyl acetate.

Antimicrobial Assay and Determination of Minimum Inhibitory Concentration (MIC and Minimum Bactericidal Concentration (MBC)

American-type culture collection of gram-positive bacteria, *Staphylococcus aureus* ATCC 43300, and gram-negative bacteria, *Klebsiella pneumoniae* ATCC 700603, *Escherichia coli* ATCC 25922 and

Shigella sonnei ATCC 25931 were used as test organisms. Test bacterium broth cultures were made, following the 0.5 McFarland standard, resulting in a final inoculum of 1.5×10^8 CFU/mL and spread over the respective MHA media using sterile cotton. Five 6 mm wells were made in the cultured media and each of the wells was then filled with 75 µL plant extract along with negative control (75 µL of 50 % DMSO) and positive control (75 µL of 1mg/mL neomycin) using a micropipette. The plates were incubated at 37°C for 24 h. The zone of inhibition on the plates was assessed, and the relevant measurements were taken in mm.

The extracts and positive control (neomycin) were diluted serially in sterile 96-well plates containing 100 µL Muller Hinton Broth (MHB) of double-strength. For each extract, two columns of 96-well plates were used. The bacterial column and media column contained only MHB. The dilution of 0.5 McFarland turbidity culture (1: 100) resulted in an ultimate concentration of 10^6 CFU/mL which were added to wells except for the media control column. After 24 h incubation of the plates at 37 °C with a sterilized lid, 5 µL of 0.003 % Resazurin solution was added to each well and kept in the incubator for 2 h at 37 °C. The well which inhibits bacterial growth remained blue while the well-turned pink due to bacterial growth. The least concentration that stopped observable growth of bacteria were considered as MIC. The evaluation of MBC was carried out by swiping the contents of the wells on nutrient agar plates and leaving them at 37 °C for more than 18 h.
